# Natural and lab-derived microbiomes differentially shape stressor interaction patterns of *Daphnia magna*

**DOI:** 10.1093/ismejo/wrae249

**Published:** 2025-05-12

**Authors:** Shira Houwenhuyse, Lore Bulteel, Isabel Vanoverberghe, Anna Krzynowek, Marlies Van de Maele, Manon Coone, Silke Van den Wyngaert, Arne Sinnesael, Robby Stoks, Ellen Decaestecker

**Affiliations:** Microbiome EcoEvo Group, Laboratory of Aquatic Biology, Department of Biology, Campus KULAK, Eteinne Sabbelaan 53, University of Leuven - KU Leuven, Kortrijk 8500, Belgium; Laboratory of Microbiology, Department of Biochemistry and Microbiology, Faculty of Sciences, Karel Lodewijk Ledeganckstraat 35, Gent University, Gent 9000, Belgium; Karel Lodewijk Ledeganckstraat 35, Ghent 9000, Belgium; Microbiome EcoEvo Group, Laboratory of Aquatic Biology, Department of Biology, Campus KULAK, Eteinne Sabbelaan 53, University of Leuven - KU Leuven, Kortrijk 8500, Belgium; Microbiome EcoEvo Group, Laboratory of Aquatic Biology, Department of Biology, Campus KULAK, Eteinne Sabbelaan 53, University of Leuven - KU Leuven, Kortrijk 8500, Belgium; Laboratory of Molecular Bacteriology, Department of Microbiology, Immunology and Transplantation, Herestraat 49, PO Box 1037, Rega Institute for Medical Research—KU Leuven, Leuven 3000, Belgium; Evolutionary Stress Ecology and Ecotoxicology, Charles Debériotstraat 32, University of Leuven—KU Leuven, Leuven 3000, Belgium; Microbiome EcoEvo Group, Laboratory of Aquatic Biology, Department of Biology, Campus KULAK, Eteinne Sabbelaan 53, University of Leuven - KU Leuven, Kortrijk 8500, Belgium; Department of Biology, Vesilinnantie 5, University of Turku, Turku 20014, Finland; Microbiome EcoEvo Group, Laboratory of Aquatic Biology, Department of Biology, Campus KULAK, Eteinne Sabbelaan 53, University of Leuven - KU Leuven, Kortrijk 8500, Belgium; Evolutionary Stress Ecology and Ecotoxicology, Charles Debériotstraat 32, University of Leuven—KU Leuven, Leuven 3000, Belgium; Microbiome EcoEvo Group, Laboratory of Aquatic Biology, Department of Biology, Campus KULAK, Eteinne Sabbelaan 53, University of Leuven - KU Leuven, Kortrijk 8500, Belgium

**Keywords:** antagonistic interaction, cyanobacteria, *daphnia*, laboratory-derived microbiome, microbiome, natural-derived microbiome, oomycete, parasite, interacting stressors

## Abstract

Organisms are facing multiple, potentially interacting stressors in natural populations. The ability of populations coping with combined stressors depends on their tolerance to individual stressors and how stressors interact, which may not be correctly captured in controlled laboratory settings. One reason for this is that the microbial communities in laboratory settings often differ from the natural environment, which could result in different stressor responses and interaction patterns. In this study, we investigated the impact of single and combined exposure to a toxic cyanobacterium and an oomycete parasite on the performance of three *Daphnia magna* genotypes. *Daphnia* individuals were sterilized and subsequently exposed to a natural or a laboratory-derived microbial inoculum. Survival, reproduction and body size were monitored, and gut microbiomes were characterized. Our study confirmed that natural and laboratory microbial inocula and gut microbiomes are differently structured. An antagonistic interaction between the two biotic stressors was revealed with respect to survival when *Daphnia,* across all three genotypes, were exposed to the laboratory microbial inoculum, with a higher survival in the multiple stressor treatment than in the single stressor treatments. In contrast, no antagonistic interaction was detected in *Daphnia* exposed to a natural microbial inoculum, where the interaction effects were mainly host genotype-dependent. Our results provide the first causal evidence that host-stressor interaction patterns may be shaped by the gut microbiome and the uptake from certain strains from the environment. This raises concern that the many multiple stressor studies on lab-cultured animals with a differently structured microbiome may provide misleading results.

## Introduction

Organisms are increasingly facing a multiple-stressor world [1–3]. There is increasing evidence that the different biotic and abiotic stressors can interact and generate complex effects on natural populations [2] that cannot be easily predicted by the effects caused by the single stressors [3, 4]. The net impact of multiple stressors can either be greater than (i.e. synergistic interaction), equal to (i.e. additive interaction), or lower than (i.e. antagonistic interaction) the sum of their single effects [1, 5]. Although synergistic interactions are expected between multiple stressors [6], a previously performed meta-analysis [1] showed that antagonistic interactions between stressors frequently occur in freshwater ecosystems. For example, warming reduced the acute toxicity of copper sulphate in channel catfish (*Ictalurus punctatus*) [7], and oxygen depletion reduced the negative effect of nickel chloride on locomotor activity in zebrafish (*Danio rerio*) [8]. Despite the crucial importance to understand and be able to predict the stressor interaction type, this remains a huge challenge and there is concern that the multiple-stressor studies done under lab conditions may not adequately capture patterns in natural populations [3, 9].

In the last decade, studies have shown that it is not only the host’s genome that determines host fitness and reaction towards stressors, but rather the complex interplay of the host genome and the microbiome [10–12]. The gut microbiota, the collection of all microorganisms present in the host’s gut, plays a key mediating role in host physiology (e.g. immunoregulation: [13]; metabolism: [14]) and host tolerance to stressors such as, e.g. parasites [15, 16] and environmental toxins [11, 17]. In addition, studies on *Daphnia*, fish and mice have shown that the gut microbiota from hosts in laboratory conditions differ in terms of microbiome structure from its free-roaming counterpart under natural conditions [18–22]. Host organisms under laboratory conditions encounter fewer microbes compared with their free-roaming counterparts, which could ultimately be reflected in (i) less diverse and/or (ii) less adapted host microbiomes. We therefore hypothesize that having a lab-derived or natural microbiome may modulate the host response to stressors, and potentially the stressor interaction pattern. Indeed, in most cases, a high bacterial diversity is a factor in protecting the host against stressors. For example, a higher soil bacterial diversity reduces the invasion of pathogens [23]. In addition, when encountering a smaller pool of environmental bacteria, the host could encounter fewer opportunities to recruit certain strains and as such obtain a less adapted host microbiome. As the host microbiome plays a crucial role in, amongst others, immune responses, exogenous exposure to laboratory microbiota could potentially not mirror expected tolerances to a given stressor as occurring in natural populations [24].

In this paper, we focused on the effect of multiple stressors on the fitness and gut microbiota of the zooplankter *Daphnia magna*. This is a key grazer in ponds and lakes worldwide, and a well-known model system to study both plastic and genetic responses to environmental stress (e.g. [25, 26]). The microbial community in *Daphnia* is structured, amongst others, by diet [27], host genetics [28–30], and cyanobacterial exposure [28]. It was demonstrated that *Daphnia* functioning is largely determined by environmental bacteria, suggesting a role of horizontally transmitted symbionts [31]. In addition, it was shown that exogenous exposure to different environmental pools of bacteria, resulted in different gut microbial communities [32]. These results show an important role of the environmental bacterioplankton community in structuring the gut microbial community in *Daphnia*. In addition, it has been suggested that the host can recruit specific strains [28, 32]. The many stressor studies on *Daphnia* have mostly used lab-derived bacterioplankton communities, which have a reduced species richness compared to natural bacterioplankton communities [32].

One important biotic stressor that is becoming increasingly dominant in aquatic ecosystems, is exposure to cyanobacteria [33–35] as they produce a wide range of toxic, secondary metabolites (e.g. cyanotoxins), among which hepatotoxins, neurotoxins and dermatotoxins [36, 37], which are hazardous to both human and livestock health. The negative effects of cyanobacteria on zooplankton performance and fitness are well documented [38–41]. Another increasing threat, especially driven by global change, are parasites [42, 43]. Fungal and mold-like parasitism received increasing scientific interest (for zooplankton: e.g. [44–46]; for cyanobacteria: e.g. [47, 48]). Fungal and mold-like infections of *Daphnia* populations occur frequently and negatively impact *Daphnia* fitness and population densities [49–53]. In addition, oomycete (i.e. fungal-like eukaryotic microorganisms) infections impact zooplankton populations in the field [54, 55] and in the laboratory [56, 57], by affecting survival and host population densities.

The increasing abundance of cyanobacterial blooms and increasing interest in mold-like parasites, sparked studies to examine potential interactions between cyanobacteria and fungi on aquatic organisms. Two research groups [58, 59] focused on chytrid (i.e. group within the fungi that produces flagellated zoospores) infections of respectively cyanobacterial populations and inedible algae and studied their effects on *Daphnia.* They showed that the presence of both stressors had a positive effect on *Daphnia* fitness compared to single stressor exposure (i.e. the toxic cyanobacteria or inedible algae). This antagonistic interaction effect on *Daphnia* fitness was obtained as: (i) *Daphnia* can consume chytrid zoospores, (ii) chytrids can break down cyanobacterial filaments, increasing its digestibility for *Daphnia*, and (iii) *Daphnia* can graze on chytrids which have obtained increased nutritional value from the infected algal cells [58, 59]. This can result in a transfer of energy and nutrients from cyanobacteria or inedible algae to zooplankton via chytrids [59, 60]. Other research focused on altered host–parasite interactions by feeding infected *Daphnia* with cyanobacteria [61, 62]. A lab experiment, revealed an antagonistic interaction between a mold-like parasite and the cyanobacteria *Microcystis aeruginosa* [62]. A higher survival was observed in infected *Daphnia* compared with non-infected *Daphnia* when fed with *M. aeruginosa*. Antagonistic interactions between different biotic stressors have also been revealed by other *Daphnia* studies (e.g. predation × parasitic bacterium: [63]; pesticide × parasitic bacterium: [64]; salinity × parasitic bacterium: [65]; cyanobacteria × parasitic iridovirus: [61]; microsporidium × parasitic bacterium: [66]; parasites × toxins [67, 68]).

In the current study, we investigated the performance of *Daphnia* in response to single and combined stressors and to what extent this depended on the presence of a natural or a laboratory-derived microbial gut community (i.e. by exogenous microbial exposures). We imposed four stressor treatments: a control treatment, exposure to the toxic cyanobacterium *M. aeruginosa*, infection with an oomycete parasite, and the combination of both *M. aeruginosa* and the oomycete infection. We tested three predictions. Firstly, we expected that the single stressor treatments would have a negative impact on fitness-related traits (survival, fecundity, and body size). In addition, we expected an antagonistic interaction between the two stressors for survival (based on [62]), i.e. a higher survival in *Daphnia* when exposed to both stressors simultaneously than when exposed to a single stressor only. Secondly, we expected a microbiome-mediated stressor tolerance, i.e. *Daphnia* receiving a natural microbial inoculum will have a higher tolerance to stressors (i.e. higher survival, fecundity, and body size) compared with *Daphnia* that received a laboratory-derived microbial community. We hypothesized that this would be reflected in (i) a differently structured gut host microbial community and/or (ii) the presence of bacterial strains involved in tolerance to the cyanobacterium and/or the infection in *Daphnia* receiving the natural inoculum compared with the laboratory-derived inoculum. As a result, we hypothesize the interaction type between the two stressors to be microbiome-mediated, i.e. antagonistic effects between the different stressors occur in the microbiome treatments which may depend on the microbiome type they were exposed to. Thirdly, we expected genotypic differences in the *Daphnia* responses to the stressors under the different microbiome exposure treatments. Previous research has revealed strong genotype effects on the gut microbial community, genotype effects on the stressor interaction type, and genotype × microbiome interactions with respect to stress tolerance in *Daphnia* [17, 28–30, 32, 69, 70].

## Materials and methods

### *Daphnia* culturing

To investigate the genotype effect, we used three genetically different *D. magna* genotypes: KNO 15.04, OM2 11.3 and T8. The KNO 15.04 genotype (further referred to as KNO) was isolated from a small pond (350 m^2^) in Knokke, at the Belgian coast (51°20′05.62”N, 03°20′53.63″E) [28, 29]. The OM2 11.3 genotype (further referred to as OM2) was isolated from a 3.7 ha inland pond located in Heverlee, in Belgium (50°51′45.0”N, 04°42′58.8″E) [71]. The T8 genotype was isolated from an 8.7 ha shallow, manmade pond, located in Oud Heverlee, Belgium (50°50′24.0”N, 04°39′40.4″E) [72]. All clonal lineages were established from resting eggs, isolated from the lake sediment. Two months before the start of the experiment, three independent iso-female lines for each genotype were cultured in separate jars for at least two generations to control for maternal effects. These iso-female lines were kept in a mixture of filtered tap and pond water in a 9:1 ratio and fed every other day with a saturating amount of the green algae *Chlorella vulgaris*. Medium (filtered tap water and pond water) was refreshed once per week. Animals were kept at a temperature of 19 ± 1°C and under a 16:8 h light:dark cycle in 2 L glass jars (at a density of 20 individuals/L). They were fed three times per week with saturating amounts of *C. vulgaris*. The first brood of the second generation was discarded, whereas eggs from the second brood were collected to obtain sterilized juveniles following previously published methods [17, 30].

### Algae culturing

*Daphnia* were fed with *C. vulgaris* (strain SAG 211–11 B), relatively good-quality food for *Daphnia* [73]. One of the stressors used in this experiment is the toxic cyanobacterial strain *M. aeruginosa* (strain PCC 7806), isolated from the Braakman reservoir in the Netherlands (51°19′22”N, 3°44′16″E) and part of the Culture Collections at Institute Pasteur (Paris, France)*. C. vulgaris* and *M. aeruginosa* were grown in Wright’s Cryptophyte (WC) medium and modified WC medium (without Tris), respectively. The algae were cultured under sterile conditions in a climate chamber at 22 ± 1°C with a light:dark cycle of 16:8 h in 2 L glass bottles, with constant stirring and aeration. Filters (0.22 μm) were placed at the input and output of the aeration system to avoid any bacterial contamination. The algae were weekly harvested in the stationary phase. The axenity of the algal cultures was checked by sequencing and plating on LB- and R2A-plates.

### Experimental design

**Figure 1:**
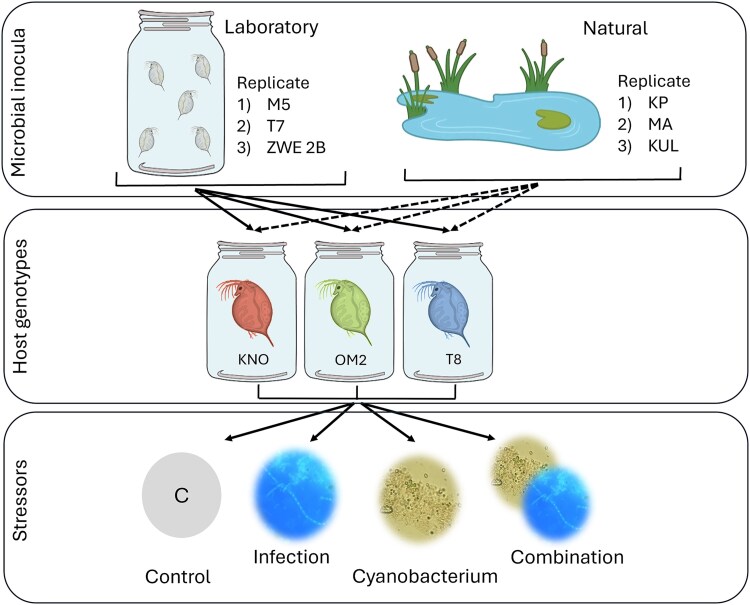
Graphic overview of the experimental design. Sterilized *daphnia* individuals from three host genotypes (KNO, OM2, T8) were exposed to a laboratory or natural-derived microbial community (microbial inocula).The experiment was performed in triplicate, with for each replicate a different laboratory and natural inoculum. Daphnia individuals receiving a laboratory-derived microbial community were exposed to 10 μm filtered tap water conditioned by *daphnia* stock cultures for one year (replicate 1: Water from cultures of daphnia genotype M5, replicate 2: Water from cultures of *daphnia* genotype T7, and replicate 3: Water from cultures of daphnia genotype ZWE 2B). *Daphnia* individuals receiving a natural microbial community were exposed to 10 μm filtered pond water (replicate 1: Water from Kennedy park pond, replicate 2: Water from Marionetten pond, and replicate 3: Water from Kulak pond). Next, the inoculated daphnia individuals were exposed to one of the four different stressor treatments: Control (C), oomycete infection, cyanobacterium or their combination. Survival and reproduction was measured for three weeks. After 21 days body size was measured, and guts were dissected.

With this experiment, we aimed to investigate the impact of the exposure of a natural versus a laboratory-derived microbial inoculum on the tolerance of *Daphnia* when exposed to two different stressors in single and combined exposures (Fig. 1). Individuals, inoculated with either a natural or a laboratory-derived microbial community, were exposed to one of the four following stressor treatments: an opportunistic oomycete parasite (further referred to as infection Fig. 1), a toxic cyanobacterium *M. aeruginosa* (further referred to as cyanobacterium Fig. 1), the combination of both the infection and the cyanobacterium, and a control treatment (fed with *C. vulgaris* instead of a mixture of *C. vulgaris* and the cyanobacterium and no exposure to the infection, further referred to as control). Each of the 24 combinations of the infection, cyanobacterium, microbial inoculum treatment and genotype was replicated independently three times using independent iso-female lines.

### Microbial inocula

All sterilized *Daphnia* juveniles received either a natural or a laboratory-derived microbial inoculum at the start of the experiment. Each microbial inoculum consisted of a water sample with a microbial community. All water samples were filtered over 100 μm and subsequently over 10 μm to remove debris. The natural microbial inocula were sampled from three natural ponds from Kortrijk. Replicate 1 received bacterioplankton from the Kennedy pond (50°48′05.7”N, 3°16′33.0″E), replicate 2 from the Marionetten pond (50°47′43.5”N, 3°15′00.2″E), and replicate 3 from the KU Leuven Kulak pond at the Ecolab (50°48′30.8”N, 3°17′37.0″E). The laboratory-derived microbial inocula were sampled from the medium in which three different *Daphnia* genotypes (different from the studied ones) had been cultured in the laboratory for more than one year. The different replicates received bacterioplankton from the culture medium of genotype M5 (replicate 1), genotype T7 (replicate 2), and genotype ZWE 2B (replicate 3). In this manner, we were able to mimic bacterioplankton communities under natural and laboratory conditions.

### Stressors

The oomycete parasite used in this experiment, was an opportunistic infection in the lab. Based on comparison with pictures [56, 57, 74], we estimated the infection to be an oomycete infection (see Figs. SI1 and SI2, and Table SI1 and SI2). This was confirmed after sequencing of samples of the infection (see supplementary information). Based on the combination of the pictures and the sequencing information, we assume that *Aphanomyces* (*Leptolegniaceae*, *Saprolegniales*, *Oomycota*) was the dominant, pathogenic species in the infection treatment. Pre-trials showed that infection with the oomycete parasite caused high mortality, especially in (germ-free) juveniles, and reduced fecundity, which appeared to differ among *Daphnia* genotypes. Infection of the oomycete parasite was visible by the presence of long, separated hyphae in biofilms on the wall of the culture jar, on the medium surface, and on dead individuals in cultures with high infection rates.

A commonly occurring and well-studied cyanobacterium is *Microcystis* sp. [75], which is known to be detrimental for *Daphnia* for many different reasons: (i) the production of various toxins, (ii) low food quality due to the absence of polyunsaturated fatty acids and (iii) colony formation which interferes with the filtering process of *Daphnia* [75–77]. In response, *Daphnia* has developed multiple tolerance mechanisms such as production of proteases or increased expression of genes associated with secondary metabolite transport and catabolism [37, 78]. *M. aeruginosa* is a colonial cyanobacterium that produces toxic metabolites such as microcystins. Many studies demonstrated negative effects of *M. aeruginosa* on *Daphnia* performance [79]. In addition, the effects of *M. aeruginosa* on *Daphnia* are genotype dependent [39], whereby the host-genotype dependent gut microbiome drives *Daphnia* tolerance to *M. aeruginosa* [28, 29].

### Stressor treatments

Individuals in the control treatment were not exposed to any stressor and were fed with *C. vulgaris* from day 3 onwards. Individuals in the oomycete infection treatment received a spore solution of lab infected individuals. The spore solution was obtained by squashing infected *Daphnia* individuals and was administered in a 1:3 ratio (1 infected, squashed, individual was used to infect three germ-free individuals in the stressor treatment). We assume little impact from the small bacterial community associated with the spore solution as the administered volume was low and as administration occurred after the exposure and colonization of the microbial inocula, for which we assumed a priority effect [32, 80]. Samples of the spore solution were sequenced to assess bacterial composition to correct for contamination if necessary. *Daphnia* in the infection treatment also received *C. vulgaris* as a food source from day 3 onwards. Individuals in the cyanobacterium treatment received a mixture of the toxic cyanobacterial strain *M. aeruginosa* and the non-toxic *C. vulgaris* in a 50:50 ratio on a daily base from day 5 onwards. Before the start of the stressor treatment (days 3 and 4), cyanobacterium-exposed individuals were fed with 100% *C. vulgaris*. Individuals in the stressor combination treatment received both the spore solution on day 5 and the combination of the toxic *M. aeruginosa* and the non-toxic *C. vulgaris* in a 50:50 ratio from day 5 onwards. Similarly, as in cyanobacterium-stressed individuals, combination-stressed individuals were fed with 100% *C. vulgaris* on days 3 and 4 (before the stressor treatment started).

### Execution of the experiment

Sterilized juveniles (0–1 day old) were individually placed in a closed vial filled with 18 ml sterile filtered tap water and 2 ml of the corresponding microbiome treatment (natural or laboratory-derived microbial community). After receiving the corresponding microbial inoculum, the individuals remained in these conditions for 48 h, allowing for the microbiota to colonize the *Daphnia* guts. On the third day, all individuals were fed with *C. vulgaris* (100*10^3^ cells/ml). On the fifth day, individuals were exposed to their corresponding stressor treatment (Fig. 1). Thereafter, the medium volume in the falcon tubes was gradually increased to 50 ml by adding 10 ml of sterile filtered tap water per day, and this for three consecutive days (day 6–8). Food concentration in the first 6 days was low (1*10^5^ cells/ml) to ensure a sufficient stress response. From day 7 onwards, food concentration was increased to 2*10^5^ cells/ml. All individuals were monitored for survival and reproduction for 21 days. At the end of the experiment (day 21), the body size was measured from top of the head to the base of the tail. The guts of the surviving *Daphnia* were dissected under a stereomicroscope with sterile dissection needles and collected per treatment in an Eppendorf tube filled with 10 μl of sterile MilliQ. This resulted in 72 gut samples, representing 2 infection treatments × 2 cyanobacteria treatments × 2 microbiome inocula × 3 genotypes × 3 replicates (Table SI3). In addition, samples of the donor microbial inocula (three natural and three laboratory-derived microbial inocula), stressor treatment (oomycete infection and *M. aeruginosa*), and food (*C. vulgaris*) were collected. Samples were stored at −20°C until further processing.

### Library preparation and sequencing

DNA was extracted using a PowerSoil DNA isolation kit (MO BIO laboratories, Carlsbad, CA, USA). DNA was dissolved in 20 μl milliQ water. Because of initially low bacterial DNA concentrations in some samples, a nested PCR was applied to increase specificity and amplicon yield. The full-length 16S rRNA gene was first amplified with EUB8F and 1492R primers on 10 ng of template using a high-fidelity SuperFi polymerase (Thermofisher, Merelbeke, Belgium) for 30 cycles: 98°C – 10 s; 50°C – 45 s; 72°C – 30 s. PCR products were subsequently purified using the CleanPCR kit (Qiagen, Antwerp, Belgium). To obtain dual-index amplicons of the V4 region, a second amplification was performed on 5 μl (=20–50 ng) of PCR product using 515F and 806R primers for 30 cycles: 98°C – 10 s; 50°C – 45 s; 72°C – 30 s. Both primers contained an Illumina adapter and an 8-nucleotide barcode at the 5′-end. For each sample, PCRs were performed in triplicate. Afterwards the PCR products were pooled, and a small volume (5 μl) was loaded on a gel to check if the PCR amplified the correct fragment. The remaining volume of the PCR products were purified using the CleanPCR kit (Qiagen, Antwerp, Belgium). An equimolar library was prepared by normalizing amplicon concentrations with a SequalPrep Normalization Plate (Applied Biosystems, Geel, Belgium) and subsequent pooling. Amplicons were sequenced using a v2 PE500 kit with custom primers on the MiSeq System (Illumina, KU Leuven Genomics Core), producing 2 × 250-nt paired-end reads.

### Analysis of *daphnia* performance traits

To assess tolerance of the *Daphnia* to the stressor treatments, we analyzed survival, fecundity and body size. Survival was analyzed using a log-rank or Mantel–Haenszel test. The survival times of individuals that were still alive at the end of the 21-day experiment were coded as right-censored. Normality and skewness of body size and fecundity data were examined with Shapiro–Wilk test, ggqqplot function (package ggpubr to make quantile-quantile plots) and Levene test. For fecundity and body size, we used the Akaike information criterion (AIC) to select the best subset of variables to represent the best model. We first evaluated to include maternal line as a random factor (with a linear mixed-effect model) or not (with a general linear model). Secondly, we tested the significance of the fixed factors in the model with the best combination of fixed and random factors according to the AIC. Type II ANOVA tables for fixed-effect terms with Satterhwaite and Kenward-Roger methods for dominator degrees of freedom for F-tests and *p*-values were created (Anova function of the car package). Following the AIC criterium, a linear mixed-effect model was chosen to evaluate fecundity and body size. In the final model, we included microbiome treatment, infection (absent or present), cyanobacterium (absent or present) and genotype as fixed factors, and maternal line as random effect. We also included all possible interactions. Post hoc analyses were performed using the “emmeans” function with a “Tukey” adjustment from the emmeans R package. All statistical tests were performed in R 4.0.2 [81].

### Analysis of *daphnia* microbiome data

DNA sequences were processed following previously published pipeline [82]. Sequences were trimmed (the first 10 nucleotides and from position 180 onwards) and filtered (maximum of two expected errors per read) on paired ends jointly. Sequence variants were inferred using the high-resolution DADA2 method, which relies on a parameterized model of substitution errors to distinguish sequencing errors from real biological variation [83]. Chimeras were subsequently removed from the data set. Taxonomy was assigned with a naïve Bayesian classifier using the SILVA v138 training set. ASVs with no taxonomic assignment at the phylum level or which were assigned as “chloroplast” or “cyanobacteria” were removed from the data set. After filtering, a total of 3 552 490 reads were obtained with on average 39038.35 reads per sample, with most samples having more than 1000 reads. To visualize the bacterial orders that differed between the treatments, ASVs were grouped at the order level, and orders representing <1% of the reads were discarded.

Measures for α-diversity of the recipient gut microbial communities within the different treatments (ASV richness) were calculated using the vegan package in R [84]. All samples were rarified to a depth of 1000 reads, based on the number of reads per sample and rarefaction curves (Fig. SI3), before analyzing α-diversity. Rarefaction was performed as there was a big difference in number of reads per sample (min: 0 reads, max 180 576 reads). The effects of sample type (donor bacterioplankton or recipient), infection (absence or presence), cyanobacterium (absent or present), and microbiome (lab and natural) treatments, genotype (KNO, OM2, and T8), and all possible interactions on ASV richness were assessed through a generalized linear model (GLM), assuming a Poisson distribution of the data and corrected for overdispersion (i.e. Quasi-Poisson). Maternal line was not included as a random factor as the AIC criterium indicated that the model without inclusion of the maternal line was a better predictive model of the data. Pairwise comparisons among significant variables and their interactions were performed using the “emmeans” function with a “Tukey” adjustment from the emmeans R package.

To examine differences in gut microbial community composition (β-diversity) among samples, Bray-Curtis, weighted and unweighted UniFrac distance matrices were calculated and plotted using Principal Coordinates Analysis with the phyloseq package in R. Multivariate community responses to treatments and genotype were investigated by means of PCA. The effect of the infection, cyanobacterium, and microbiome treatments, genotype, and all possible interactions on β-diversity were assessed through a permutation MANOVA, using the Adonis2 function in the vegan package in R. Obtained *P*values were adjusted for multiple comparisons through the control of the false discovery rate (FDR).

Pearson correlations were executed between the number of sequenced guts and the ASV richness to check for interdependence. No significant correlation could be detected between the number of sequenced guts and ASV richness, dismissing the issue of interdependence (Table SI4). Additionally, correlation tests were executed between the three life history traits and the ASV richness of the gut microbial communities. Obtained *p*-values were adjusted for multiple comparisons through the control of the FDR.

To identify which bacterial orders significantly differed between the treatments, relative abundances per order were calculated on the raw sequencing data, excluding the samples removed by the rarefaction. The uptake of bacteria by the recipient *Daphnia* from the donor bacterioplankton, was also analyzed with Union plots using the wilkox/unionplot function from GitHub (https://github.com/wilkox/unionplot; results see supplementary information, Fig. SI4). Additionally, differential abundance analyses were performed (edgeR function) on the raw sequencing data from which samples with less than 2 counts per million (CPM) in at least three samples were filtered out. All statistical tests were performed in R 4.0.2 [81].

## Results

### Performance traits of the *daphnia* host

We investigated the impact of a natural versus a laboratory-derived microbial community on the tolerance of *Daphnia* exposed to an oomycete infection (characterization of the oomocyete infection, see supplementary information) and/or the toxic cyanobacterium *M. aeruginosa* using three *Daphnia* genotypes (KNO, OM2, T8) with three replicates per treatment combination. Sterilized *Daphnia* of each genotype received a natural (pond water) or a laboratory-derived (culturing medium) microbial inoculum and were exposed to one of the four stressor treatment combinations for 21 days: control treatment (no stressor exposure), the oomycete infection (exposure to a spore solution from infected individuals), the cyanobacterium *M. aeruginosa*, or the combination of both stressors. To assess *Daphnia*’s tolerance to the stressor (s), we monitored individual survival and fecundity (total number of hatched eggs per *Daphnia*) during the 21-days stressor exposure period and measured their body size (from top of the head to base of the tail) at the end of the stressor exposure period.

**Figure 2:**
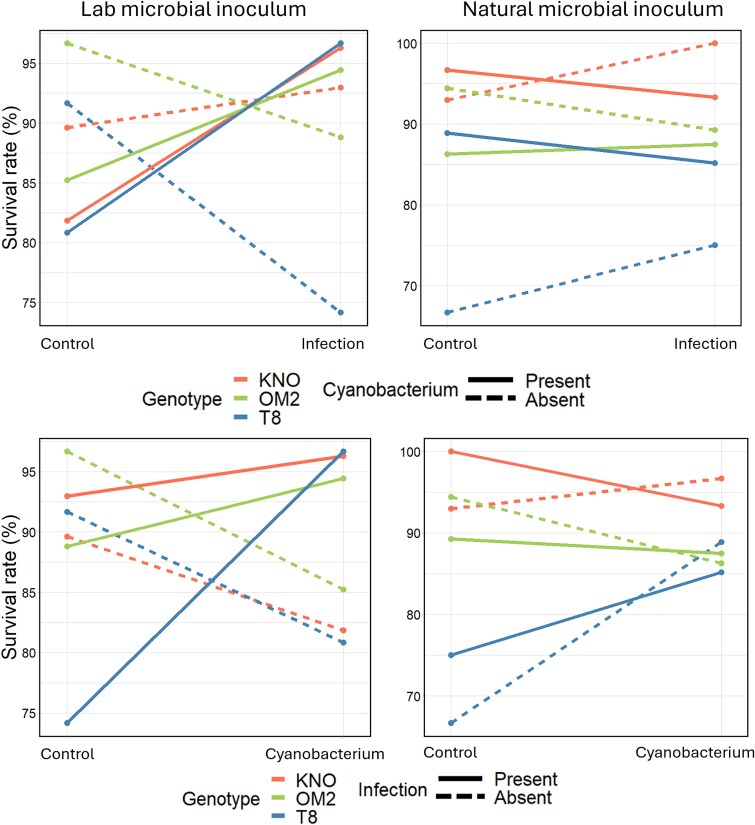
Interaction plot for the effects of the oomycete infection and the cyanobacterium on the survival rate of *daphnia* individuals grouped per microbiome treatment. Left column: Lab microbial inoculum: *Daphnia* individuals that received a laboratory-derived microbial inoculum, right column: Natural microbial inoculum: *Daphnia* individuals that received a natural microbial inoculum. The colors represent the different genotypes: KNO (red), OM2 (green) and T8 (blue). The lines show the reaction norms in the presence (full line) or absence (dotted line) of the cyanobacterium (upper row) or the infection (bottom row). Both the upper and bottom row show the same data, only arranged in a different way. On the upper row, the infection is on the x-axis and the presence/absence of the cyanobacterium is shown with the full/dotted lines. On the bottom row, the cyanobacterium is on the x-axis and the presence/absence of the oomycete infection is shown with full/dotted lines.

**Figure 3:**
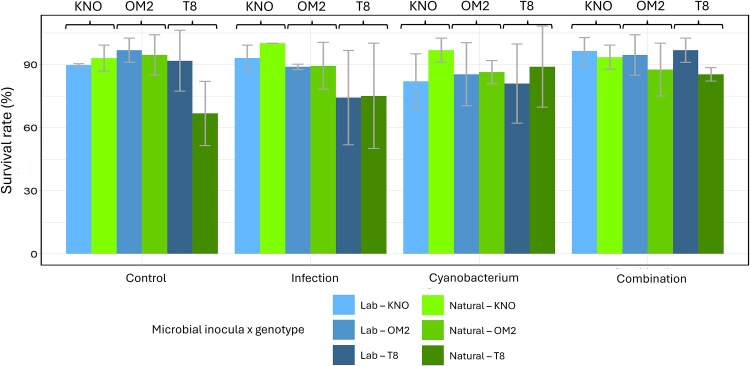
Bar plot of the survival rate of *daphnia* individuals grouped per stressor treatment on the x-axis. Labels above the bar stand for the genotype: K = KNO, O = OM2 and T is T8. *Daphnia* individuals inoculated with a laboratory microbiome (blue: light, medium, and dark). *Daphnia* individuals inoculated with a natural microbiome (green: light, medium, and dark). Error bars represent one standard deviation of the data.

The survival analysis revealed an oomycete infection × cyanobacterium × microbial inoculum × genotype interaction on *Daphnia* survival (Table 1A). To further investigate this four-way interaction, we analyzed the data for the lab and natural microbial inocula separately (results are shown in Table SI5). When *Daphnia* received a laboratory-derived microbial inoculum, there was a significant infection × cyanobacterium interaction (X^2^ = 9.5, df = 3, *P* = 0.02) reflecting the antagonistic interaction between the two stressors on *Daphnia* survival (Fig. 2). We found that (Figs. 2 and 3) when exposed to a laboratory-derived inoculum, survival was higher (i) in the control treatment than when exposed to the cyanobacterium (X^2^ = 4.9, df = 1, *P* = 0.03; on Fig. 2, left column, bottom row, all dotted lines decline, which means that the cyanobacterium treatment has a lower survival than the control treatment), but not when exposed to the infection (X^2^ = 1.9, df = 1, *P* = 0.2; on Fig. 2, left column, top row, two of the three dotted lines decline, which means that the oomycete infection had for two of the three genotypes a lower survival than the control treatment) and (ii) when exposed to both stressors than exposed to a single stressor (infection: X^2^ = 3.5, df = 1, *P* = 0.05; cyanobacterium: X^2^ = 6.9, df = 1, *P* = 0.009; on Fig. 2, left column, all full lines, representing the multiple stressor treatment, are higher than the dotted lines which represent the single stressor treatments). When *Daphnia* received a natural microbial inoculum, the interaction between the stressor treatments was genotype dependent (infection × cyanobacterium × genotype interaction, X^2^ = 22, df = 11, *P* = 0.02, Table SI5). There was not a significant antagonistic interaction between the two biotic stressors for any of the genotypes when averaged over both microbiome types (KNO: X^2^ = 2.6, df = 3, *P* = 0.5; OM2: X^2^ = 4.4, df = 3, *P* = 0.2; T8: X^2^ = 2.3, df = 3, *P* = 0.5; Table SI5). To summarize, an antagonistic interaction was present between the two stressors for survival, but only when *Daphnia* received a laboratory microbial inoculum.

**Table 1:** Overview results life history traits and gut microbiome community (ASV richness and beta diversity based on unweighted Unifrac distance) using LMER.

**A**	**Survival**			**Fecundity**			**Body Size**			**ASV richness**			**Beta diversity**	
	**df**	***P* value**	**Chi** ^**2**^	**df**	***P* value**	***F***	**df**	***P* value**	***F***	**df**	***P* value**	***F***	***P* value**	***R*** ^**2**^
	**Recipient *Daphnia***													
**Infection**	1	0.3	0.9	1, 589.00	0.001^*^	9.6593	1, 204.00	<0.001^***^	19.3880	1, 0.000	0.99	0	0.227	0.026
**Cyanobacterium**	1	1	0	1, 589.07	<0.001^***^	129.3836	1, 204.01	<0.001^***^	121.3782	1, 1.446	0.42	0.68	0.301	0.023
**Microbiome**	1	0.8	0	1, 589.01	0.035^*^	4.4413				1, 18.715	0.007^*^	8.84	0.001^*^	0.145
**Genotype**	2	0.009^*^	9.4	2, 589.74	<0.001^***^	30.0048				2, 12.399	0.76	2.93	0.470	0.041
**Microbiome** × **Genotype**	5	0.02^*^	13.9	2, 589.05	0.077	2.5756				2, 1.053	0.78	0.24	0.865	0.031
**Infection × Genotype**	5	0.05	11.1	2, 589.03	0.318	1.1479				2, 5.636	0.28	1.33	0.727	0.034
**Cyanobacterium × Genotype**	5	0.02^*^	13.2	2, 589.03	<0.001^***^	9.8391				2, 5.858	0.27	1.38	0.496	0.041
**Infection × Microbiome**	3	0.8	1.1	1, 589.07	0.379	0.7749				1, 1.385	0.42	0.65	0.507	0.020
**Cyanobacterium × Microbiome**	3	0.9	0.4	1, 589.02	0.254	1.3057				1, 2.895	0.25	1.36	0.323	0.023
**Infection × Cyanobacterium**	3	0.4	3.3	1, 589.06	0.274	1.1980	1, 204.00	0.1138	2.5216	1, 7.493	0.07	3.54	0.246	0.025
**Infection × Microbiome × Genotype**	11	0.1	16.9	2, 589.12	0.041^*^	3.2062				2, 3.690	0.43	0.87	0.171	0.061
**Cyanobacterium × Microbiome × Genotype**	11	0.05	19.9	2, 589.09	0.519	0.6557				2, 15.363	0.04^*^	3.63	0.775	0.033
**Infection × Cyanobacterium × Genotype**	11	0.07	18.8	2, 589.12	0.235	1.4519				2, 9.650	0.12	2.28	0.729	0.034
**Infection × Cyanobacterium × Microbiome**	7	0.2	10.2	1, 589.09	0.657	0.1976				1, 0.160	0.78	0.07	0.359	0.022
**Infection × Cyanobacterium × Microbiome × Genotype**	23	0.04^*^	36.3	2, 589.02	0.015^*^	4.2544				1, 1.011	0.49	0.47	0.470	0.020
**B**	**Survival**		**Fecundity**		**Body Size**		**ASV richness**			**Beta diversity**				
	***P* value**	**Chi** ^**2**^	***P* value**	***F***	***P* value**	***F***	**df**	***P* value**	***F***	***P* value**	***R*** ^**2**^			
**Donor bacterioplankton**														
**Microbiome**							1, 47.682	<0.001^***^	1.69	0.021^*^	0.262			

Table 1A presents the results for *Daphnia* recipients, while table 1B shows the results for the bacterioplankton donors. Sample type refers to the origin of the sample, i.e. donor bacterioplankton or recipient gut *Daphnia*. Significant results (*P* < 0.05) are indicated with ^*^. Highly significant results (*P* < 0.001) are indicated with ^***^. df = degrees of freedom.

**Figure 4:**
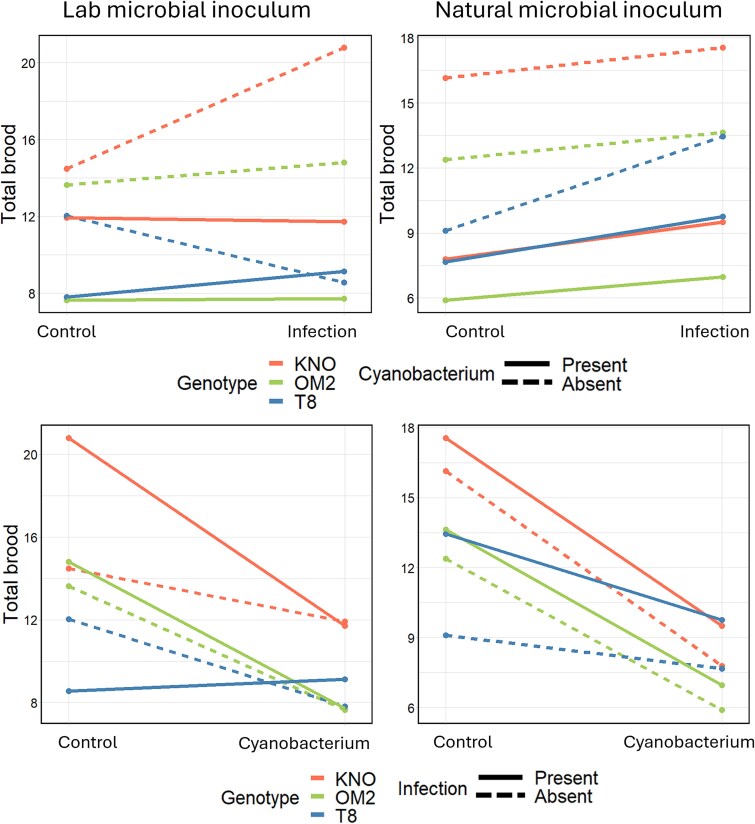
Interaction plot for the effects of the oomycete infection and the cyanobacterium on the total fecundity in *daphnia* grouped per microbiome treatment. Left column: Lab microbial inoculum: *Daphnia* individuals that received a laboratory-derived microbial inoculum, right column: Natural microbial inoculum: *Daphnia* individuals that received a natural microbial inoculum. The colors represent the different genotypes: KNO (red), OM2 (green) and T8 (blue). The lines show the reaction norms in the presence (full line) or absence (dotted line) of the cyanobacterium (upper row) or the infection (bottom row). Both the upper and bottom row show the same data, only arranged in a different way. On the upper row, the infection is on the x-axis and the presence/absence of the cyanobacterium is shown with the full/dotted lines. On the bottom row, the cyanobacterium is on the x-axis and the presence/absence of the oomycete infection is shown with full/dotted lines.

**Figure 5:**
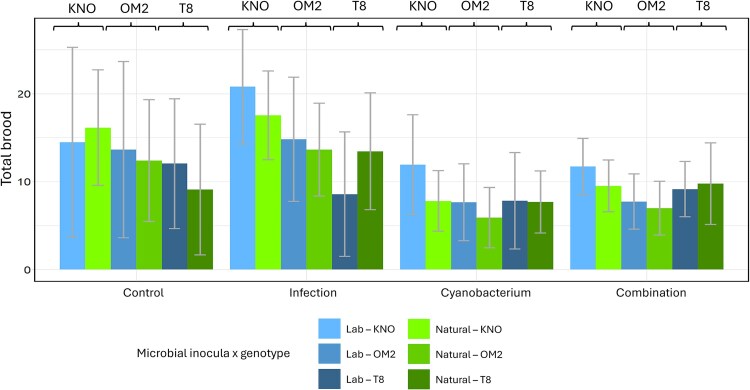
Bar plot of the total fecundity of the *daphnia* individuals grouped per stressor treatment on the x-axis. Labels above the bar stand for the genotype: K = KNO, O = OM2 and T is T8. *Daphnia* individuals inoculated with a laboratory microbiome (blue: light, medium, and dark). *Daphnia* individuals inoculated with a natural microbiome (green: light, medium, and dark). Error bars represent one standard deviation of the data.

The analyses on total fecundity (total number of hatched eggs per *Daphnia* individual) revealed a four-way oomycete infection × cyanobacterium × microbiome × genotype interaction (Table 1A). Separate analyses per microbial inoculum treatment (statistical results in Table SI6) indicated that fecundity was reduced for all three genotypes (KNO: *F* = 77.6, df = 1215.04, *P* < 0.0001; OM2: *F* = 69.5, df = 1206.04, *P* < 0.0001, T8: *F* = 5.8, 1, 164.67, *P* = 0.01) when exposed to the cyanobacterium in both microbial inoculum treatments (laboratory: *F* = 42.5, df = 1292.02, *P* < 0.0001, natural: *F* = 126.4, df = 1295.02, *P* < 0.0001; Figs. 4 and 5). When exposed to the infection, fecundity increased, particularly when *Daphnia* received a natural microbial inoculum (for two of the three genotypes in the laboratory microbial inoculum treatment: *F* = 3.1, df = 2292.05, *P* = 0.04, and for all three genotypes in the natural microbial inoculum treatment: *F* = 13.8, df = 1295.02, *P* = 0.0002). For fecundity, no significant infection × cyanobacterium interaction (antagonistic interaction) was detected, neither when exposed to the lab microbial inoculum (*F* = 1.2, df = 1292.01, *P* = 0.28) or the natural microbial inoculum (*F* = 0.8, df = 1295.04, *P* = 0.38). In summary, the type of microbial inoculum only had an effect on fecundity when *Daphnia individuals* were exposed to the oomycete infection (not when exposed to cyanobacteria or the multiple stressor treatment), and this effect was genotype dependent.

**Figure 6:**
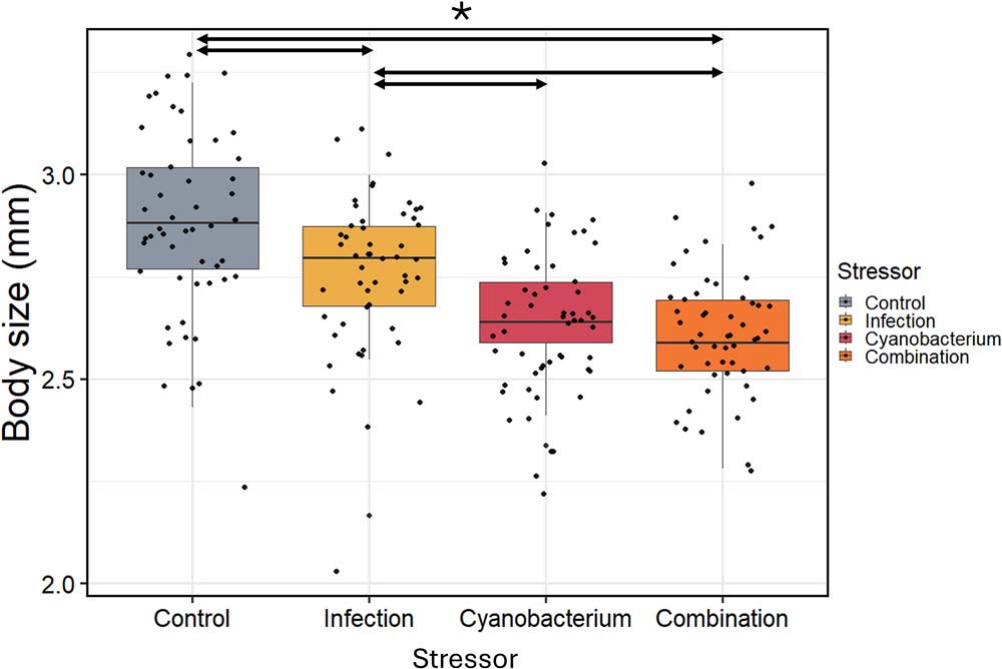
Box plots of the body size of *daphnia* at the end of the experiment per stressor treatment. Colors indicate the different stressor treatments. Black dots represent the individual data points. Arrows above the boxplots represent significantly different treatments.

Analyses on *Daphnia* body size at the end of the stressor exposure period revealed significant effects of the single infection and cyanobacterium treatments, but not of their interaction. Effects of the stressor treatments on body size, did not depend on *Daphnia* genotype or microbial inoculum (Table 1A, Fig. 6). Post hoc analyses showed a significant difference between all stressor treatments, except between the single stressor cyanobacterium and the stressor combination treatment (Table SI7). Individuals in the control treatment had the highest body size, followed by, in decreasing order of body size, individuals exposed to the infection, cyanobacterium and the stressor combination treatment, from which the last two did not significantly differ, suggesting an antagonistic interaction between the two stressors on body size (Fig. 6). The type of microbial inoculum had no effect on body size.

### Composition of the microbiome communities

To analyze the gut microbial community composition, the guts of the surviving *Daphnia* after the 21-days stressor exposures period were collected and the V4 region of the 16S rRNA gene was sequenced (total of 72 gut samples, representing 2 oomycete infection treatments × 2 cyanobacteria treatments × 2 microbiome inocula × 3 genotypes × 3 replicates). In addition, the bacterial community composition of the donor microbial inocula (3 natural and 3 laboratory-derived microbial inocula), the medium used for the stressor treatments (oomycete infection and the cyanobacterium *M. aeruginosa*) and food (*C. vulgaris*) was determined. The laboratory microbial inocula were dominated by *Micrococcales* (40.6%), *Burkholderiales* (23.3%) and *Chitinophagales* (16.2%), whereas the natural microbial inocula were dominated by *Burkholderiales* (31.3%), *Kapabacteriales* (25.1%), *Sphingobacteriales* (9.2%), *Flavobacteriales* (7.2%), and *Chitinonphagales* (6.2%). In the recipient *Daphnia* microbial communities, the same four bacterial orders were the most abundant in each treatment combination and genotype (Table 2). The observed top four taxa in the *Daphnia* guts consisted of: *Burkholderiales*, *Pseudomonadales*, *Verrucomicrobiales*, and *Rhizobiales*. Relative abundance tables can be found in the supplementary information (Table SI8).

**Table 2:** Relative abundance of the top four most dominant bacterial orders in the recipient gut microbiomes, grouped per microbiome treatment, stressor treatment and genotype.

Order	Lab microbiome	Natural microbiome	Infection absent	Infection present	Cyanobacterium absent	Cyanobacterium present	KNO	OM2	T8
Burkholderiales	57.00	41.17	49.48	49.84	47.87	51.68	46.77	44.75	57.32
Pseudomonadales	12.46	34.73	23.26	22.26	24.67	20.68	27.81	24.61	15.22
Verrucomicrobiales	5.83	5.25	5.13	6.10	4.80	6.44	4.81	6.59	5.58
Rhizobiales	5.27	4.00	4.26	5.22	6.80	2.24	5.81	7.11	1.22

#### ASV richness

**Figure 7:**
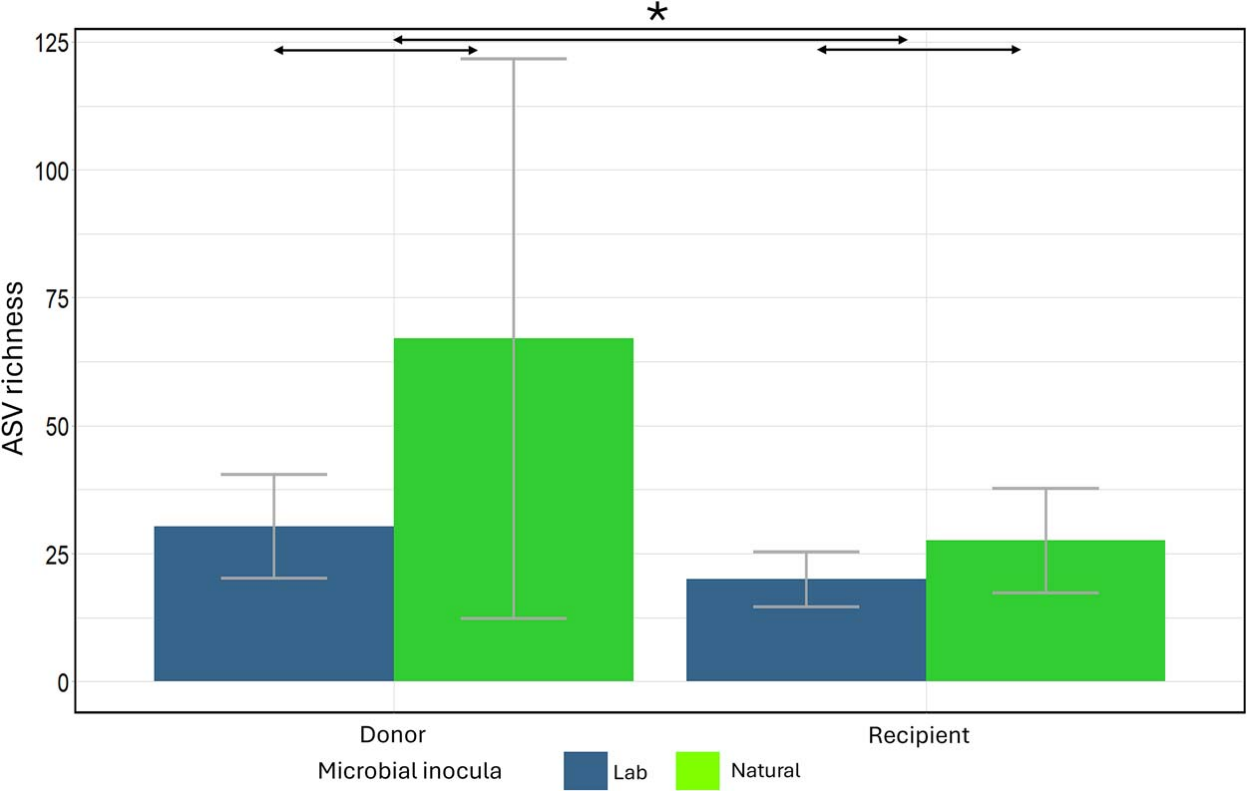
Bar plots of ASV richness of donor inoculum and recipient *daphnia* samples grouped per sample type (donor inoculum vs recipient daphnia) and microbial inocula. Colors indicate the different microbial inocula. Error bars indicate one standard error.

As a measure for α-diversity of the samples, the ASV richness was determined. In both donor inocula and recipient gut microbiomes, ASV richness was significantly higher in the natural conditions compared with the laboratory conditions (Table SI9, Fig. 7). ASV richness was also significantly higher in the microbial inocula compared with the gut microbiomes of the recipient *Daphnia* (*P* < 0.001, z-value = −12.13, Fig. 5), which suggests a selective recruitment of bacteria in the *Daphnia* gut. Analysis of the recipient *Daphnia* revealed a significant microbial inoculum effect and a significant cyanobacterium × microbial inoculum × genotype interaction on ASV richness (Table 1A). A separate analysis per microbial inoculum treatment did not reveal a significant main effect of the cyanobacterium treatment in *Daphnia* that received a natural microbial inoculum (infection: *F* = 0.1, df = 1,0.37, *P* = 0.75; cyanobacterium: *F* = 0.9, df = 1,3.15, *P =* 0.36). *Daphnia* that received a laboratory-derived microbial inoculum showed marginally significant interactions between infection and genotype or cyanobacterium treatment (infection × genotype: *F* = 3.3, df = 2,6.7, *P* = 0.07 and infection × cyanobacterium: *F* = 4.0, df = 1, 4.1, *P* = 0.07). No correlations were observed between the *Daphnia* performance traits and ASV richness of the gut microbial community (Table SI10, Fig. SI5). In conclusion, in *Daphnia* that received a laboratory microbial inoculum, ASV richness was marginally influenced by the infection, either in interaction with the other stressor or with the *Daphnia* genotype.

#### Beta diversity

**Figure 8:**
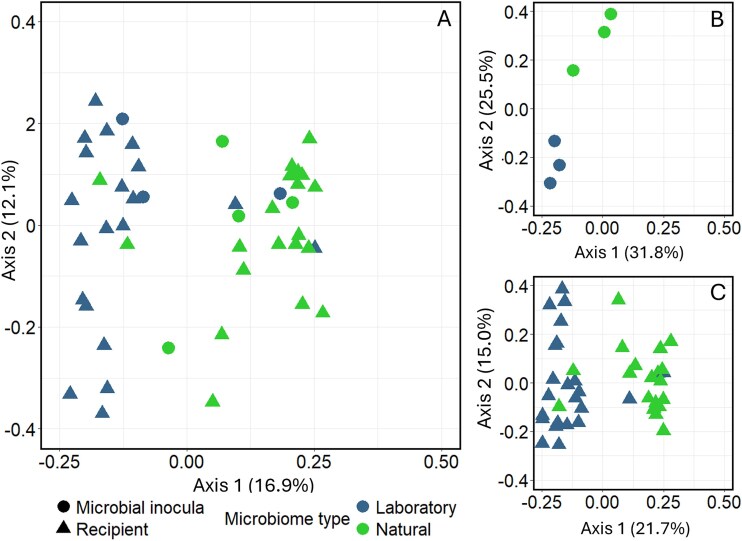
PCoA showing significant differences between natural and laboratory-derived microbial inocula and recipient gut microbiomes (A), the microbial inocula (B), and the resulting *daphnia* gut microbiomes (C), based on the unweighted UniFrac distances. Shapes represent the different microbiome types (circle: Microbial inocula, triangle: Recipient gut microbiomes). Colors represent the two microbial inoculum types: Laboratory microbial communities (blue), and Natural microbial communities (green).

The beta diversity analyses (PERMANOVAs) on the microbial donor inocula revealed a significant difference between the natural and laboratory-derived microbial inocula, based on unweighted UniFrac distances (Table 1B, Fig. 8A). Analyses on beta diversity on the gut microbial composition revealed that *Daphnia* receiving the natural microbial inoculum differed significantly from those receiving the lab microbial inoculum based on unweighted UniFrac distances (Table 1A, Fig. 8B). None of the stressor treatments, genotypes or interactions were significant (Table 1A). Separate analyses per microbial inoculum treatment did not reveal a significant main effect of the infection, cyanobacterium or the genotype or any significant interaction in the gut microbiome composition of *Daphnia* that received a laboratory-derived or a natural microbial inoculum based on unweighted UniFrac distances. In summary, the type microbial inoculum did not affect the beta-diversity when looking at the stressor treatments, genotypes, or their interactions.

#### Microbial community

The differential abundance (EdgeR) analysis revealed highly significant differences for 213 ASVs between the donor bacterioplankton and the recipient *Daphnia* (Table SI11). Within the donor bacterioplankton, only the relative abundance of one ASV (*Aurantimicrobium* sp. Family *Microbacteriaceae*) was significantly different between the laboratory-derived and the natural microbial inocula (Table SI11, the three laboratory-derived microbial inocula were pooled and the three natural microbial inocula were pooled). Within the recipients the relative abundance of 141 ASVs were significantly different between the four stressor treatments, the relative abundance of 285 ASVs between the microbial inoculum treatments, the relative abundance of 34 ASVs differed when stressor and microbial inoculum treatments were combined and the relative abundance of 5 ASVs differed when stressor, microbial inoculum treatment and *Daphnia* genotype were combined (Table SI11; Fig. SI6). Analysis per microbial inoculum treatment revealed that the relative abundance significantly differed between the stressor treatments for 12 ASVs within the laboratory-derived microbial inoculum treatment and for 24 ASVs within the natural microbial inoculum treatment (Table SI11, Fig. SI7). A summary of significant differences in the relative abundance of the most abundant ASVs between the stressor treatments per microbial treatment can be found in Table 3. This table shows that the relative abundance of the family *Microbacteriaceae* was significantly higher in the infection-cyanobacteria combination treatment when *Daphnia* received a laboratory-derived microbial inoculum, compared to when *Daphnia* received a natural microbial inoculum or when they were not exposed to a stressor treatment. In addition, this table also shows that the relative abundance of this ASV was higher in the gut microbial community of *Daphnia* that received the laboratory-derived versus the natural microbial inoculum.

**Table 3:** Relative abundance of the top thirteen most dominant ASVs in the recipient gut microbiomes, grouped per microbiome treatment, infection (absent or present) and cyanobacterium (absent or present).

**OTU**	**Lab**	**Natural**	**Infection absent**	**Infection present**	**Cyanobacterium absent**	**Cyanobacterium present**	***P* value lab**	***P* value natural**
ASV_2_Polynucleobacter_sp.	38.28	24.62	33.30	30.19	27.85	36.62	No	No
ASV _1_Pseudomonadaceae	11.26	33.69	22.07	21.21	24.32	18.68	No	No
ASV _3_Burkholderiales	13.95	9.46	9.94	14.30	11.75	12.00	No	No
ASV _7_Rhizobiaceae	3.89	3.44	3.69	3.67	5.32	1.79	No	No
ASV _6_Streptococcus_sp.	3.81	1.27	2.46	2.84	3.10	2.09	3.89604E-07	1.09817E-05
ASV _10_Luteolibacter_sp.	0.66	3.74	0.99	3.49	2.68	1.43	No	No
ASV _14_Comamonadaceae	2.60	1.57	3.11	0.87	3.87	0.12	No	No
ASV _4_Microbacteriaceae	3.02	0.83	1.77	2.30	1.93	2.09	3.89604E-07	1.17556E-06
ASV _5_Verrucomicrobiaceae	3.13	0.45	2.81	0.72	1.10	2.79	No	1.89948E-06
ASV _9_Burkholderiales	0.02	3.34	0.76	2.58	2.86	0.08	No	No
ASV _24_Flavobacterium_sp.	0.74	1.50	1.20	0.95	1.29	0.86	1.36035E-07	No
ASV _8_Verrucomicrobiaceae	1.85	0.27	1.10	1.14	0.43	1.91	No	7.27336E-06
ASV _16_Chitinophagales	1.36	0.48	1.47	0.29	1.31	0.54	No	No

The last two columns represent the results from the EdgeR analysis, showing significant differences between the stressor treatments (stressor treatments were grouped) when exposed to a laboratory-derived or a natural bacterial inoculum.

## Discussion

We investigated the performance of *Daphnia* in response to single and combined stressors (i.e. toxic cyanobacterium, oomycete infection, or a combination of those two), and to what extent this is dependent on the presence of a natural or a laboratory-derived microbial community. We hypothesized that: (i) single stressors would negatively impact *Daphnia* fitness and that there would be an antagonistic interaction between the stressors for survival; (ii) *Daphnia* receiving a natural microbial inoculum would have a higher tolerance to stressors than *Daphnia* receiving a laboratory microbial inoculum and that the interaction type between the two stressors would be microbiome-mediated; (iii) *Daphnia* responses to the stressors and microbiome treatments would be genotype-dependent. We found that natural and laboratory-derived microbial inocula were differently structured, as were the resulting gut microbial communities in the recipient hosts, with the natural microbiomes having a higher ASV species richness than the laboratory microbiomes. Next, we showed that exposure to cyanobacteria reduced survival, fecundity and body size, whereas exposure to the oomycete infection only reduced body size. Effects on survival and fecundity were also dependent on the microbial inoculum type. The interaction between the oomycete infection and cyanobacterium depended on the microbial inoculum type: the oomycete infection and cyanobacterium interacted antagonistically on survival when *Daphnia* were exposed to a laboratory-derived microbial inoculum, but not when they were exposed to a natural microbial inoculum. Finally, we revealed *Daphnia* responses to the stressors and microbial inocula treatments were genotype dependent for survival and fecundity, but not for body size.

Consistent with our first prediction, the natural microbial inocula had a higher ASV richness and were differently structured than laboratory-derived microbial inocula, and this was also reflected in the gut microbiomes of the recipient *Daphnia*. These results confirm the findings of two other *Daphnia* studies [22, 32], which reported a general trend, where *Daphnia* exposed to treatments with a higher environmental bacterial α-diversity showed a higher bacterial α-diversity in the gut microbiome; most likely due to the presence of a larger pool of potential colonizers. Differently structured bacterial communities in natural versus laboratory environments were also observed for other hosts (e.g. *Drosophila* [85], *Limulus polyphemus* [86], zebrafish [18], mice [20]).

All three performance traits, survival, fecundity, and body size, were reduced when exposed to the cyanobacterial stressor, whereas only body size was reduced when exposed to the oomycete infection. For survival and fecundity these effects were dependent on the microbial inoculum (supporting our second prediction) and the recipient *Daphnia* genotype. This is consistent with the cyanobacterium *M. aeruginosa* being detrimental for *Daphnia* fitness [38–41]. Based on personal observations of this virulent oomycete infection in the laboratory, we also expected negative effects of the infection on *Daphnia* survival and fecundity. This was based on the observation of high mortality in stock cultures when the infection was present in the laboratory, also upon the presence of infected eggs in the brood pouch, and low hatching success after sterilization of infected eggs. The lower impact of the oomycete infection compared to the cyanobacterium may stem from the used genotypes in this experiment. As they survived previous outbreaks of the infection in the laboratory, they may display relatively low susceptibility to the infection.

A key finding was that the expected antagonistic interaction between the two stressors was only observed on the survival data, and only when the *Daphnia* received a laboratory-derived microbial inoculum and not when they received a natural microbial inoculum. It was shown that an antagonistic interaction between mold-like infections and cyanobacteria for *Daphnia* survival exist in laboratory experiments [58, 59, 62, 68]. This antagonism was explained by the observation that obligate, fungal parasites from inedible diatoms and cyanobacteria, can transfer energy and nutrients from otherwise inedible algae to infection used here also infects cyanobacteria [58, 59]. However, we assume chances are low as aquatic oomycetes strains generally infect animal and plant host species, whereas no widespread evidence of oomycetes infecting cyanobacteria is known. Furthermore, cyanobacteria are used as a means of biocontrol in agricultural crops to suppress fungal and oomycete phytopathogens [87], reducing chances that the cyanobacterium in our experiment is infected by the used oomycete infection. The obtained results are in line with our second hypothesis: that tolerance to the stressors and the stressor interaction type is microbiome-mediated. Only infected individuals experienced a (genotype-dependent) microbiome-effect for fecundity. Previous research already suggested a strong genotype-effect of the uptake and structuring of the gut microbial strains. In addition, it is possible that this result only comes forward in the oomycete infection (and not in de cyanobacteria or combination treatment) as the microbiome has a more significant impact on reproductive health during parasitic infections than during cyanobacterial stress due to the direct nature of infections, localized immune responses, and resource competition that parasites induce.

*Daphnia* survival and fecundity were, thus, differently impacted when they received the natural or the laboratory-derived microbial inoculum, however body size was not affected by the microbiome inocula. There are several hypotheses available why body size was not affected by microbiome treatment. A first hypothesis can be found in the process of how parasites and hosts allocate resources and the biological priorities of the host under stress (e.g. by cyanobacteria, infection, or combination). In many cases, survival and reproductive success are prioritized over growth because they are more critical for the host's evolutionary fitness. Secondly, the intensity of parasitic infection had an effect on body size, however, survival, or fecundity are more commonly impacted. In mild or moderate infections, hosts may tolerate the parasite well, showing little effect on body size but still experiencing reductions in reproductive output or longevity. As the infection in our experiment did not result in strong fitness effects, we expect that infections with higher parasite loads or stronger fitness effects, may result in a more pronounced impact on body size.

The dependence of the interaction type on the microbial inoculum type can be explained as the natural microbial community was differently structured, and could provide a broader pool of microbiota. Originally, we expected this broader pool of bacteria to be beneficial for *Daphnia* fitness. A broader pool of bacteria is beneficial if (i) a general uptake of more strains includes strains that have a positive effect on defense mechanisms linked with survival, or (ii) if *Daphnia* recruits microbial strains to protect the host against the stressor. Nevertheless, we found that when *Daphnia* were inoculated with a laboratory-derived microbial inoculum, the antagonistic effect between the stressors appeared. In that combination, the relative abundance of one family, the *Microbacteriaceae*, was significantly higher. This could potentially result from reduced competition between multiple bacterial strains when bacterial diversity is lower (i.e. bacterial richness is lower), such that beneficial strains (e.g. *Microbacteriaceae*) can thrive better as previously hypothesized [88]. Bacterial strains in guts with low bacterial diversity (i.e. low bacterial richness) may interact more intensively with the stressors or boost host tolerance indirectly more strongly (as they do not have to compete with many other bacterial strains). *Microbacteriaceae* are bio-degraders capable of producing hydrolytic enzymes, such as chitinase, cellulase, and glucanase [89, 90]. Part of these components are present in cyanobacteria and oomycetes. As such, the presence of this group of bacteria could have increased the availability of essential nutrients and substrates for the *Daphnia* individuals, which in turn could have contributed to the increased *Daphnia* survival [89]. The causal role of *Microbacteriaceae* in increasing *Daphnia* survival under combined stressor treatments needs, however, further investigation, e.g. through mono-association experiments. Antagonistic effects between symbionts (other than bacteria) have been detected in *Daphnia* as well, e.g. between microsporidia and fungal infections [16], and between microsporidia or cyanobacteria and a viral infection [61, 66]. A broader pool of microbiota can also reduce host survival, if next to beneficial and neutral microbial strains, opportunistic negative strains are taken up. Aquatic environments contain, next to a plethora of beneficial and neutral microbial strains, obligate and opportunistic pathogens [90, 91], so it could be that with a higher microbial diversity more opportunistic, pathogenic strains were present as well [27]. We did, however, not find a correlation between gut microbial diversity and the observed *Daphnia* performance traits, which suggests that the diversity of the gut community did not predominantly determine tolerance in *Daphnia* here.

Whatever the exact underlying mechanism, the finding that the interaction type between two stressors can be mediated by the gut microbiome adds a new perspective to multiple-stressor research. There is increasing concern and research on combined stressor effects and particularly on how stressors interact as this may crucially determine their impact on natural populations [3, 9]. Our ability to predict the interaction type is, however, still very limited, and improved insights in the determinants of the interaction type are therefore highly needed. Our finding that the microbial inoculum can change the interaction type between the two biotic stressors tested and that this is linked with the presence of microbiota strains, even for the same host genotype, suggests that the gut microbiome may be an important determinant to consider in future studies to understand the occurrence of stressor interaction patterns. Moreover, the finding that a natural gut microbiome may cause a different stressor interaction pattern than a lab-derived gut microbiome adds an extra dimension for the call to work with natural microbiomes [24]. Extrapolating multiple stressor studies from the lab to the field remains a huge challenge and our results indicate that one ignored factor is that many multiple-stressor studies used lab-reared organisms with a differently structured microbiome than field organisms.

We also found support for the hypothesis that interaction patterns among stressors can be genotype-dependent, which moreover was also microbiome-dependent. This extends the more widely documented pattern that stress responses, as here in terms of survival and fecundity, can be host genotype-dependent. These single stressor results are in accordance with the literature as responses to cyanobacteria (e.g. [28, 39]) and parasites (e.g. [71, 92]) in *Daphnia* are generally considered to be genotype-dependent. In contrast, genotype effects on the interaction between stressors are not often documented. Two other studies, both on *D. magna*, compared the combined effect among genotypes of two stressors: an insecticide and another stressor (a parasite [64] and a heat spike [70]). They both showed that the interaction between the two stressors differed among genotypes. Unique to our study is that this genotype-dependence of the stressor interaction pattern was only present when the *Daphnia* received a natural microbial inoculum. *Daphnia* genotypes differ in their selective capacities to take up bacteria [28, 30, 32, 93], which in turn, can result in different responses to the stressor treatments. Some *Daphnia* genotypes will be highly selective, and e.g. take up bacteria that help in the protection against toxic cyanobacteria, whereas other *Daphnia* genotypes will be less selective and randomly take up bacterial strains from the environment. It was shown that a local adaptation effect for this response, with genotypes selecting more consistently for the same, diverse group of bacterial strains upon sympatric microbial exposure, which was not the case if bacterial strains of the microbiome were allopatric [17].

In conclusion, by using an experimental manipulation of the microbial inoculum we could provide causal evidence that the gut microbiome can not only determine the host’s tolerance to stressors but also the interaction type between stressors. Moreover, by manipulating the host exposure to a lab-derived versus a natural derived microbiome, our results underscore that the gut microbiome may be an important factor underlying differences in stressor interaction patterns between lab studies and natural populations. This indicates that the gut microbiome may be an important determinant to consider in future studies to improve our still limited understanding of the occurrence of stressor interaction patterns.

## Supplementary Material

SupplementaryInformation_ISMEj_Final_NoTrackChanges_wrae249

Table_S1_ISMEj_wrae249

Table_SI3_wrae249

Table_SI5_wrae249

Table_SI6_wrae249

Table_SI8_wrae249

Table_SI11_wrae249

## Data Availability

The datasets and scripts generated for this study can be found in the NCBI, under accession number PRJNA731313, on Zenodo with DOI: 10.5281/zenodo.4778716, and on GitHub: https://github.com/AMK06-1993/Daphnia-Magna-Oomycytes.
