# Supplementary material for: Natural and lab-derived microbiomes differentially shape stressor interaction patterns of Daphnia magna
Source: ISME J. 2025 May 12;19(1):wrae249. doi: 10.1093/ismejo/wrae249 (PMC12225683; doi:10.1093/ismejo/wrae249)
Supplement: SupplementaryInformation_ISMEj_Final_NoTrackChanges_wrae249 [file supplementaryinformation_ismej_final_notrackchanges_wrae249.docx]

**Supplementary information**

**Characterization of the oomycete infection**

***Visual characterization of the infection***

The infection is visible in living *Daphnia*, especially in juveniles as hyphae that interfere with the filter apparatus and other internal structures of the *Daphnia*. In early stages, the *Daphnia* can swim, but as infection gets worse, hyphae are also covering the carapax, reducing their capability to swim to the point that they are fully covered by the hyphae. As the *Daphnia* cannot move (are being trapped) and filter anymore, they die. We noticed evidence of infecting the hemolymph by preparing squash samples of infected *Daphnia*, infected *Daphnia* were visibly not of good health and showed a reduced fecundity in grave infection (deterioration of eggs in brood pouch).

Based on microscopic pictures of the samples of the original experiment and comparison with pictures in Prowse 1954, Green 1974 and Seymour et al. 1984, we estimate the infection to be an oomycete infection (Figure SI1).


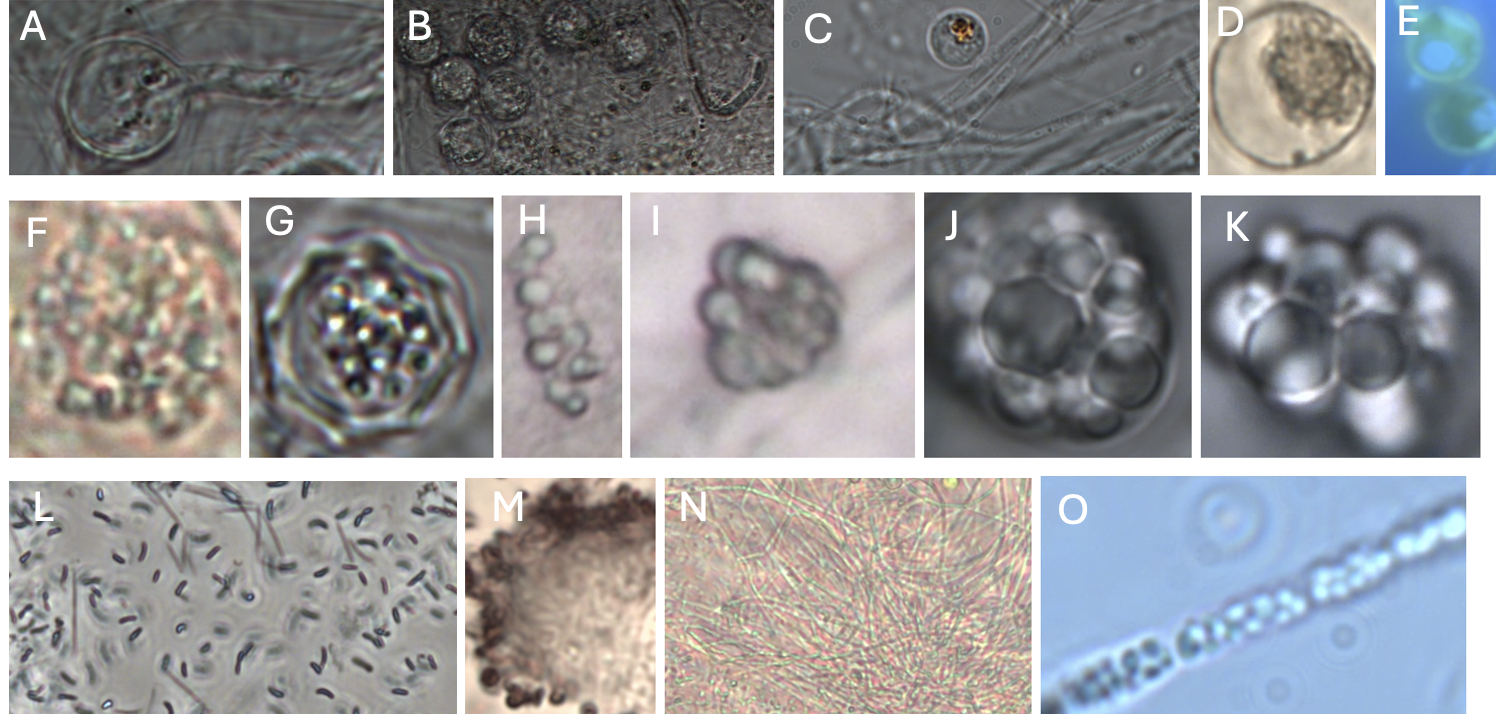


**Figure SI1**. Pictures of oomycete-like components in infected *Daphnia* microscopic pictures selected out of a collection of the sample set that has been sequenced (see further) upon the presence of *Aphanomyces*. Selection was done based on candidate pictures of different *Aphanomyces* stages in Prowse (1954), Green (1974), and Seymour et al. (1984). Sexual reproduction: oogonium (female), antheridia (male) forming oospores; asexual reproduction: zoospores in sporangia. First row: A: developing oogonium, B: developed oogonia with oospores; C-D: oogonium as cyst with oospores on top associated with non-septate hyphae, E: coloured developed oogonia. Second row: F-G: clustered oospores in oogonia without and with visible antheridia, H-K: clustered zoospores. Third row: J-M: free zoospores, N: non-septate hyphae; O: connected and clustered zoospores in sporangia.

***Whole genome shotgun metagenomics to characterize the infection***

**Sample preparation and sequencing:** To characterize the oomycete infection, several samples (table SI1) from stock cultures that showed similar oomycete presence as the ones of the experiment were sequenced. DNA was extracted using the plant/fungi isolation kit from Norgen, except for sample S2, which was extracted with the Powersoil Pro from Qiagen, and 100 ng of DNA was sent for Illumina whole genome shotgun metagenomics sequencing.

**Table SI1:** Samples used for whole genome shotgun metagenomics.

| **Sample** | **Content** |
| --- | --- |
| S1 | Medium from infected jar (2019) |
| S2 | Medium from infected jar with *Daphnia* clone B7 |
| S3 | Medium from infected jar (2020) |
| S4 | Biofilm from infected jar (2020) |
| S5 | Infected *Daphnia* from other lab |
| S6 | Biofilm from infected jar with *Daphnia* clone F |
| S7 | 10 infected *Daphnia* clone F |

**Processing of the metagenome data:** Demultiplexed paired-end reads were processed for quality control using FastQC, then trimmed and filtered with fastp to remove low-quality reads (Phred score <30) and adapter sequences, using default settings (Chen et al., 2018). This preprocessing yielded 166 659 694 high-quality sequences, ranging from 20 884 562 to 2 419 764 sequences per sample. Reads from *Daphnia magna* microbiome samples were then decontaminated by mapping to the *Daphnia magna* genome from RefSeq (NCBI) (GCF_020631705.1) using bowtie2, with default settings (Langmead and Salzberg, 2012). Unmapped reads were extracted using samtools (Li et al., 2009) and assembled into contigs with MEGAHIT (Li et al., 2015). Contigs were annotated using the CAT annotation tool (von Meijenfeldt et al., 2019), and ORFs and proteins predicted with Prodigal (Hyatt et al., 2010). Abundances were quantified using FeatureCounts (Liao et al., 2014) and custom scripts. Subsequent analyses filtered out non-target proteins, retaining those classified as Oomycetes, which were then subjected to BLAST searches against the NCBI nr database with an e-value threshold of <1e-3, keeping only the top 5 hits per search.

**Results**:

Whole genome shotgun metagenome sequencing resulted in 166 659 694 high-quality sequences, ranging from 20 884 562 to 2 419 764 sequences per sample. After quality trimming and removing the host genome (*Daphnia magna*) contamination, the resulting eukaryotes common to all infected samples were narrowed down to 20 eukaryotic clades (Table SI2). Based on microscopic pictures of the samples of the original experiment and comparison with pictures in Prowse 1954, Green 1974 and Seymour et al. 1984, we estimate the infection to be an oomycete, more specifically an *Aphanomyces* sp. infection. In the metagenome data, the Oomycota class was present, confirming the estimation based on the microscopic pictures. The samples contained on average 569.29 reads annotated as “Oomycota”. The extracted proteins of the “Oomycota” clade were identified as *Aphanomyces astaci* (58 hits), *Aphanomyces euteiches* (53 hits), and *Aphanomyces stellatus* (27 hits). Therefore, we estimate that the infection used in the experiment is likely to be an *Aphanomyces* sp.. Given that we worked with squashed, infected *Daphnia* individuals, other contaminating fungi, ciliates and bacterial sequences were present. Nevertheless, the combination of the sequences with the positive hit for *Aphanomyces* in combination with the pictures where the *Aphanomyces* like structures were very abundant (as they were at the time of the experiment) we assume that *Aphanomyces* was the dominant, pathogenic species in the infection treatment.

**Table SI2:** Eukaryotes that are present in all samples. Abundances are shown per sample. The abundance of each ORF (open reading frame) are the number of reads mapped, but adjusted for sequencing dept.

|  | S1 | S2 | S3 | S4 | S5 | S6 | S7 |
| --- | --- | --- | --- | --- | --- | --- | --- |
| Eukaryota (superkingdom) | 550018 | 4140 | 21679 | 150474 | 7027 | 72003 | 2013 |
| Oligohymenophorea (class) | 394288 | 385 | 5035 | 91231 | 235 | 34 | 179 |
| Daphnia magna (species) | 541 | 132037 | 490 | 3795 | 6201 | 98436 | 92000 |
| Hyalella azteca (species) | 52073 | 237596 | 24 | 28 | 809 | 32 | 144 |
| Tetrahymena thermophila SB210* (strain) | 105148 | 60 | 3825 | 38954 | 1554 | 15 | 89 |
| Bilateria (clade) | 29653 | 105 | 8399 | 37385 | 155 | 164 | 23 |
| Opisthokonta (clade) | 38089 | 90 | 2765 | 12858 | 156 | 1832 | 96 |
| Daphnia pulex (species) | 25789 | 4379 | 753 | 11515 | 5603 | 1338 | 5117 |
| Eumetazoa (clade) | 17951 | 387 | 5102 | 22032 | 401 | 499 | 770 |
| Stylonychia lemnae (species) | 11243 | 14089 | 2520 | 1323 | 15711 | 21 | 314 |
| Alveolata (clade) | 31341 | 191 | 728 | 7280 | 179 | 12 | 114 |
| Sar (clade) | 33067 | 92 | 627 | 4949 | 83 | 816 | 17 |
| Protostomia (clade) | 13443 | 16 | 4218 | 16856 | 7 | 112 | 503 |
| Chlorella variabilis (species) | 442 | 12338 | 192 | 496 | 61 | 3120 | 16 |
| Daphnia (genus) | 72 | 5068 | 52 | 42 | 4 | 2371 | 5739 |
| Fungi (kingdom) | 6238 | 5 | 211 | 2737 | 26 | 70 | 5 |
| Metazoa (kingdom) | 3972 | 10 | 350 | 1285 | 38 | 28 | 21 |
| **Oomycota (class)** | **3645** | **14** | **42** | **88** | **72** | **102** | **22** |
| Trebouxia sp. A1-2 (species) | 52 | 42 | 33 | 3194 | 56 | 8 | 10 |
| Tanacetum cinerariifolium (species) | 8 | 7 | 30 | 224 | 56 | 31 | 3 |

**References**

Chen, S., Zhou, Y., Chen, Y., & Gu, J. (2018). fastp: an ultra-fast all-in-one FASTQ preprocessor. Bioinformatics, 34(17), i884-i890.

Green, J. (1954). Size and reproduction in *Daphnia magna* (Crustacea: Cladocera). Proc. Zool. Soc. Lond., 124, 535-545.

Hyatt, D., Chen, G.L., Locascio, P.F., Land, M.L., Larimer, F.W., & Hauser, L.J. (2010). Prodigal: prokaryotic gene recognition and translation initiation site identification. BMC Bioinformatics, 11, 119.

Langmead, B., & Salzberg, S.L. (2012). Fast gapped-read alignment with Bowtie 2. Nature Methods, 9, 357-359.

Li, H., Handsaker, B., Wysoker, A., Fennell, T., Ruan, J., Homer, N., ... & Durbin, R. (2009). The Sequence Alignment/Map format and SAMtools. Bioinformatics, 25(16), 2078-2079.

Li, D., Liu, C.M., Luo, R., Sadakane, K., & Lam, T.W. (2015). MEGAHIT: an ultra-fast single-node solution for large and complex metagenomics assembly via succinct de Bruijn graph. Bioinformatics, 31(10), 1674-1676.

Liao, Y., Smyth, G.K., & Shi, W. (2014). featureCounts: an efficient general purpose program for assigning sequence reads to genomic features. Bioinformatics, 30(7), 923-930.

Prowse, G.A. (1954). *Aphanomyces daphnia* sp.nov., parasitic on *Daphnia hyaline.* Transactions of the British Mycological Society 37 (1), pp. 22-28.

Seymour, R., Cowgill, U.M., Klecka, G.M., Gersich, F.M., Gersich M.A. (1984). Occurrence of *Aphanomyces daphniae* infection in laboratory cultures of *Daphnia magna*. Journal of Invertebrate pathology 43(1), pp. 109-113.

von Meijenfeldt, F.A.B., Arkhipova, K., Cambuy, D.D., Coutinho, F.H., & Dutilh, B.E. (2019). Robust taxonomic classification of uncharted microbial sequences and bins with CAT and BAT. Genome Biology, 20(1), 217.

**Uptake of bacterial strains from the environment in the *Daphnia* gut**

The uptake of bacteria by the recipient *Daphnia* from the environment (donor bacterioplankton), was analyzed with Unionplots. Two Unionplots were made, one for each microbiome type. In each Unionplot, the donor bacterioplankton was compared with the gut microbiomes from the *Daphnia* that received a control treatment or a stressor treatment (Figure SI6). When *Daphnia* received a laboratory derived donor inoculum, they took up 35.8% of the donor inoculum. In the control treatment, 41.0% of the gut microbiomes consists of ASVs present in the donor laboratory bacterioplankton, while in the stressor treatments, only 34.5% of the gut microbiomes consists of ASVs present in the donor laboratory bacterioplankton. When *Daphnia* received a natural derived donor inoculum, they took up 31.4% of the donor inoculum. The difference in uptake between *Daphnia* that received a control or stressor treatment was smaller when they received a natural derived donor inoculum than when they received a laboratory derived donor inoculum. In the control treatment, 32.5% of the gut microbiomes consists of ASVs present in the donor natural bacterioplankton, and in the stressor treatments, 31.6% of the gut microbiomes consists of ASVs present in the donor natural bacterioplankton.

**Supplementary figures**


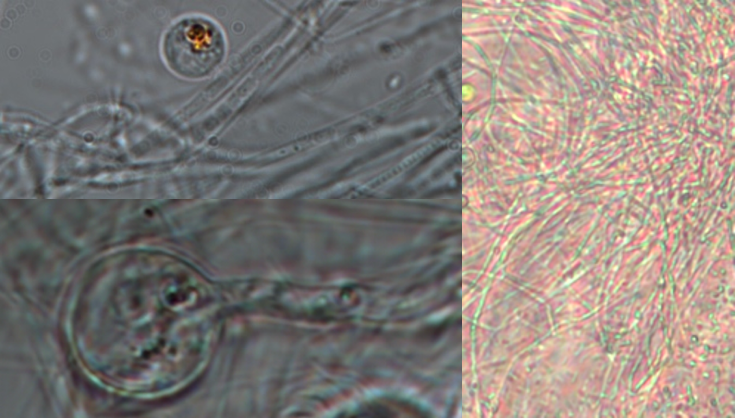

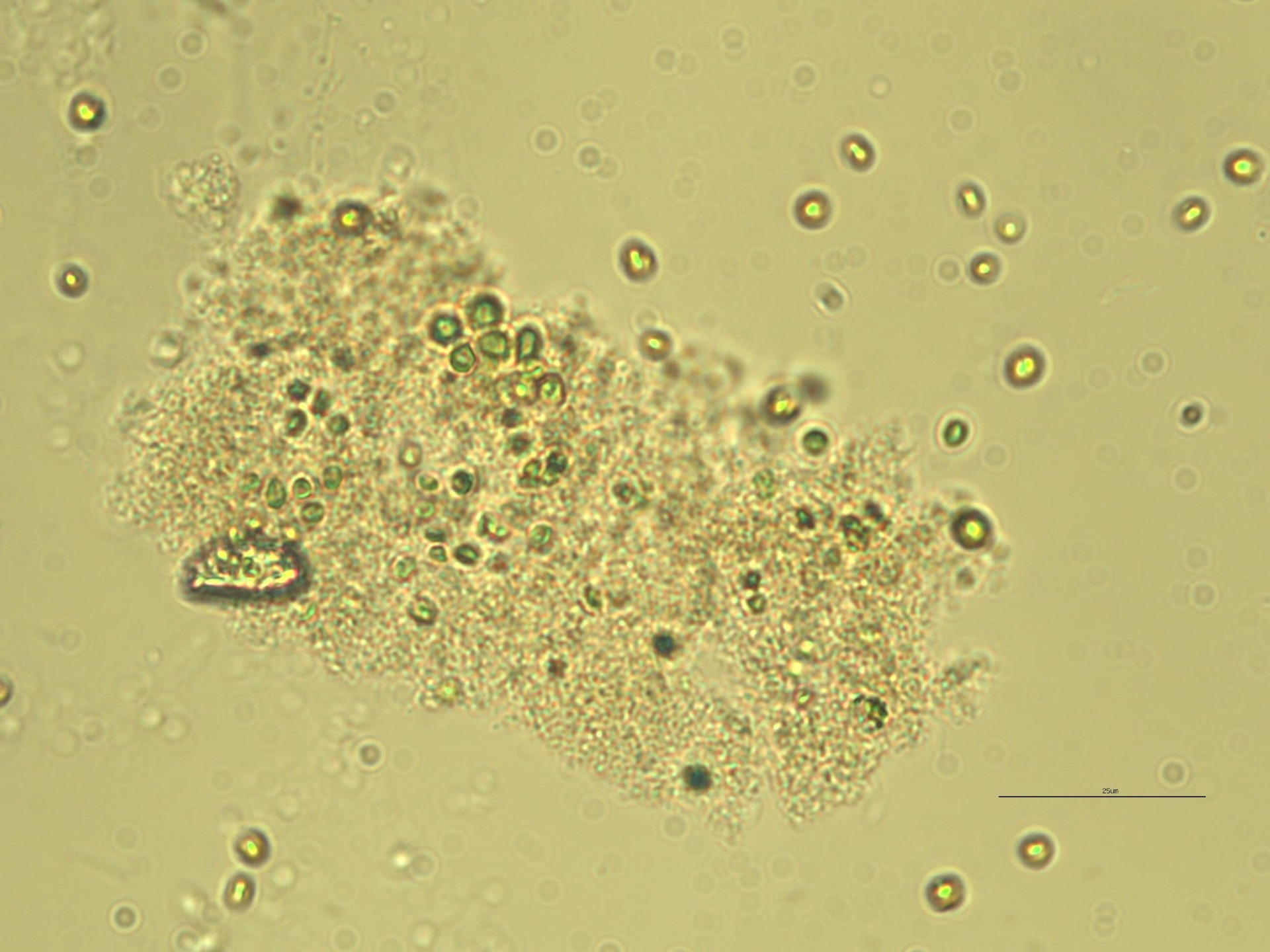


A

B

**Figure SI2**: Microscopic pictures of the stressor treatments; (A) Oomycete infection treatment: hyphae and oogonia and (B) *Microcystis* treatment: Colony of *Microcystis* surrounded with individual cells with 160 x magnification.

| 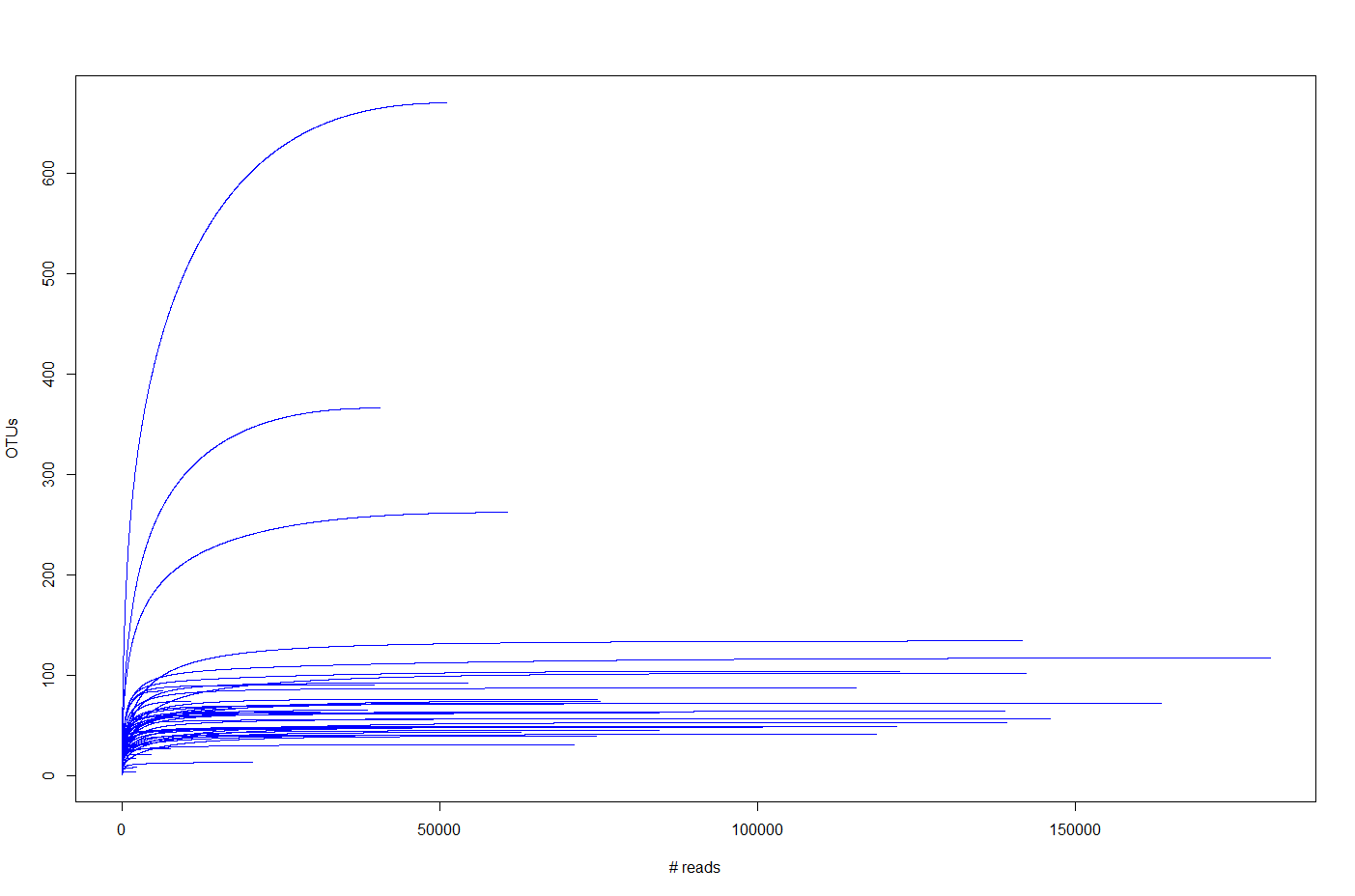 | 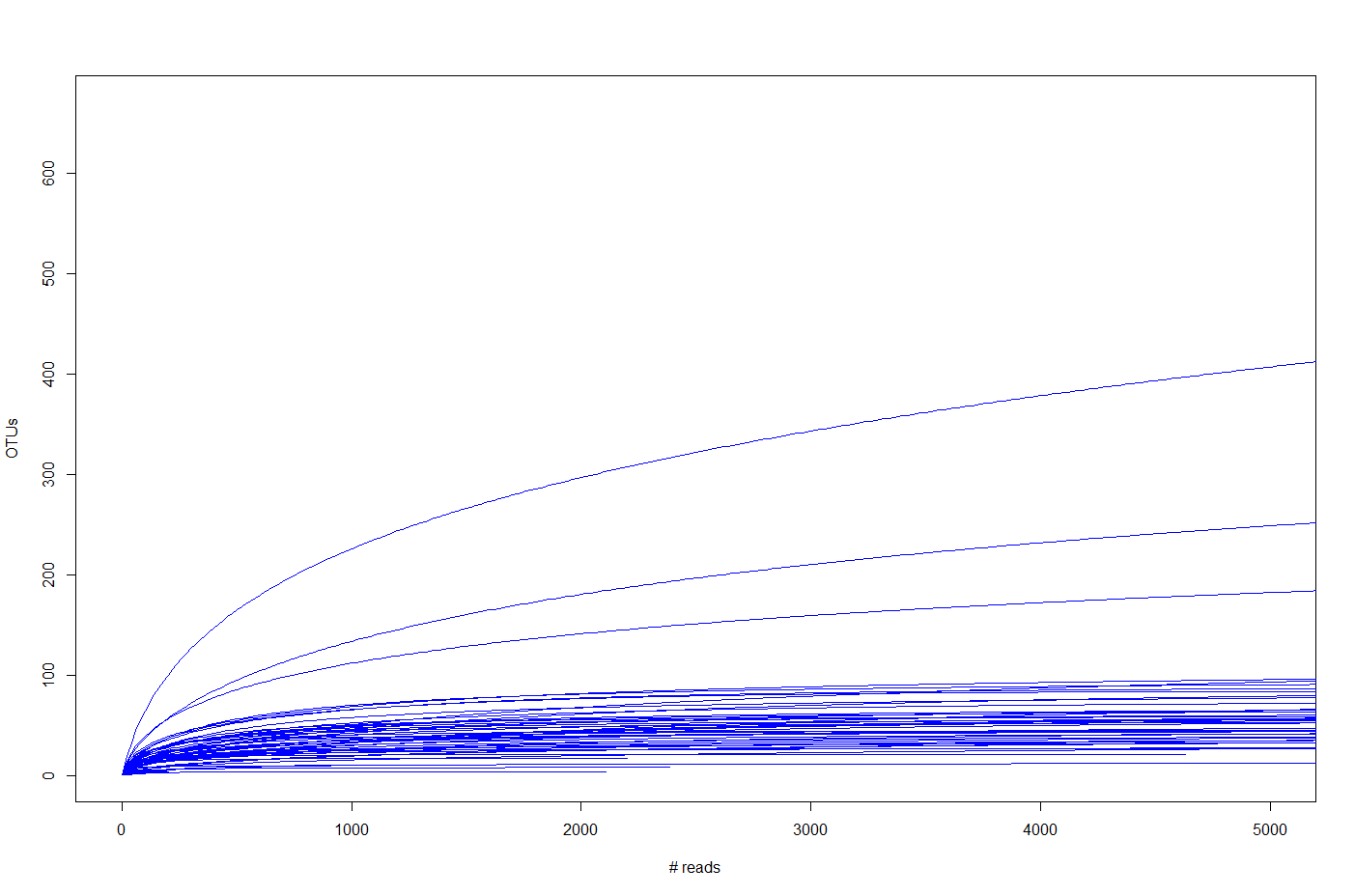 |
| --- | --- |
| A | B |

**Figure SI3**: Rarefaction curve of the raw sequencing data. Number of reads are represented on the x-axis, number of ASVs are represented on the y-axis. (A) overview (B) zoomed in on the first 5000 reads.


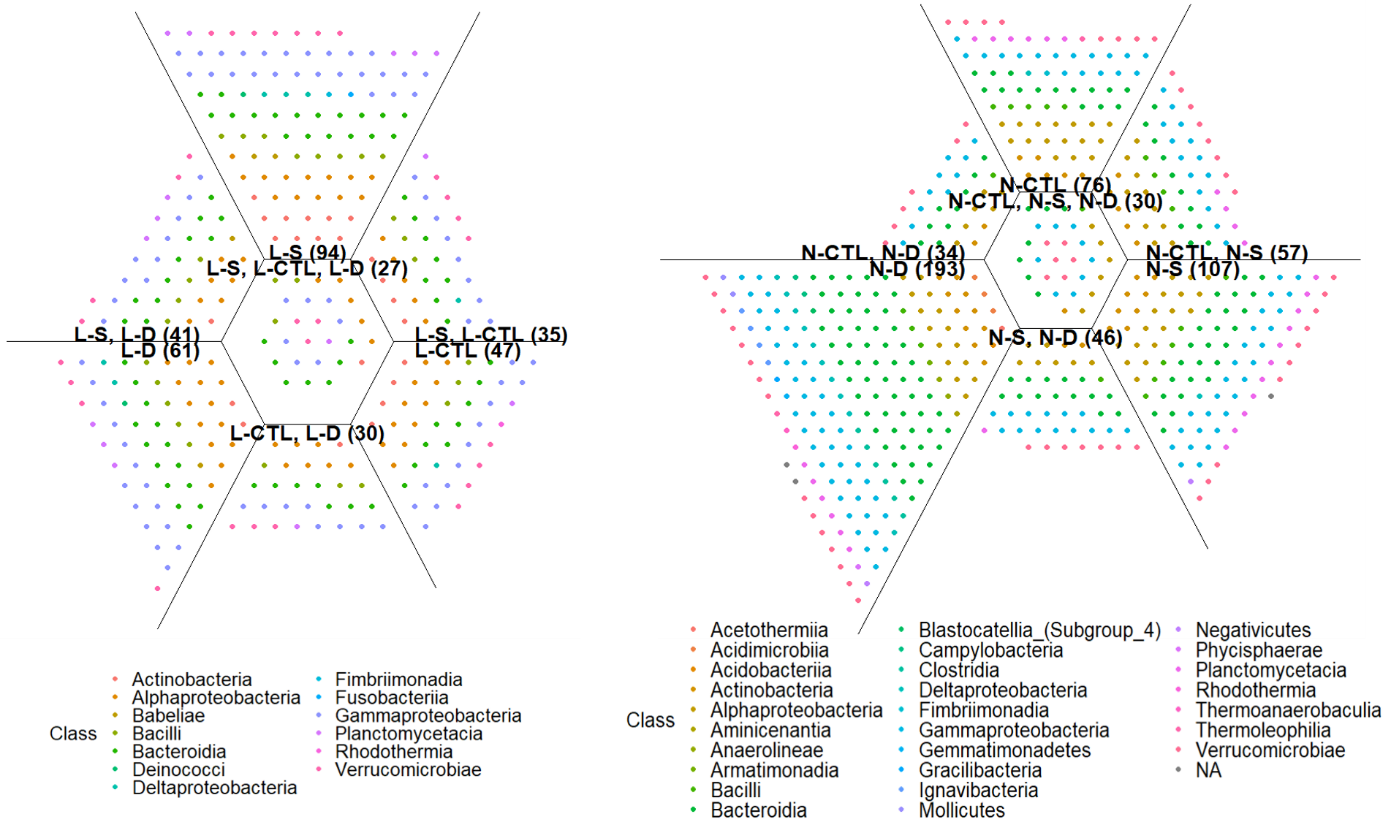


**Figure SI4**: Unionplots representing the unique and shared ASVs between donor bacterioplankton and recipient *Daphnia* in the (A) laboratory treatment and (B) natural treatment. L=laboratory treatment, N=natural treatment, S=stressor treatment (infection, cyanobacterium and combination), CTL=control treatment, D=donor bacterioplankton.


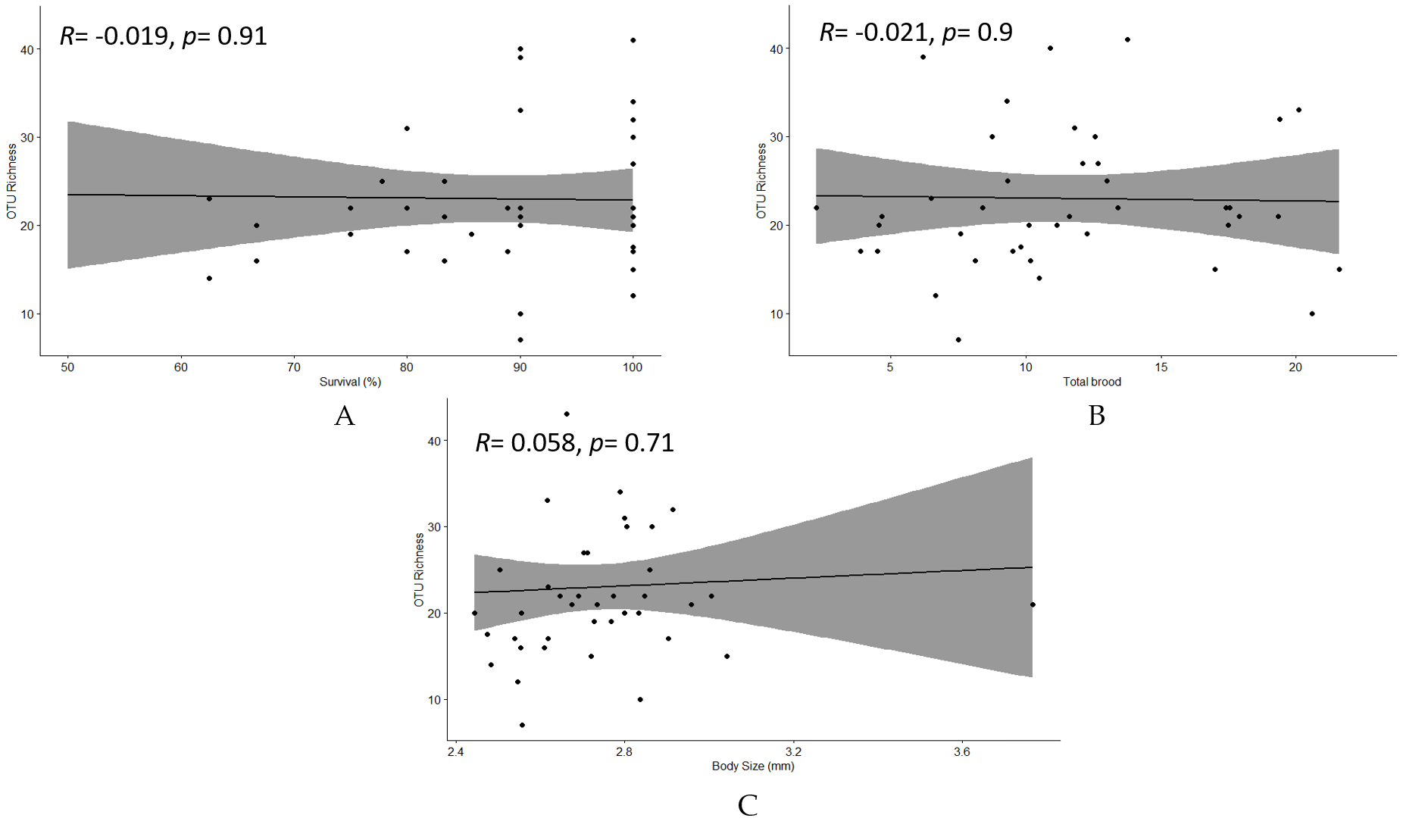


**Figure SI5**: Pearson regression between (A) Survival, (B) Fecundity, (C) Body size and ASV richness of the gut microbial community of recipient *Daphnia*. Non-adjusted p-values and correlation coefficient (R) are noted per figure.

| 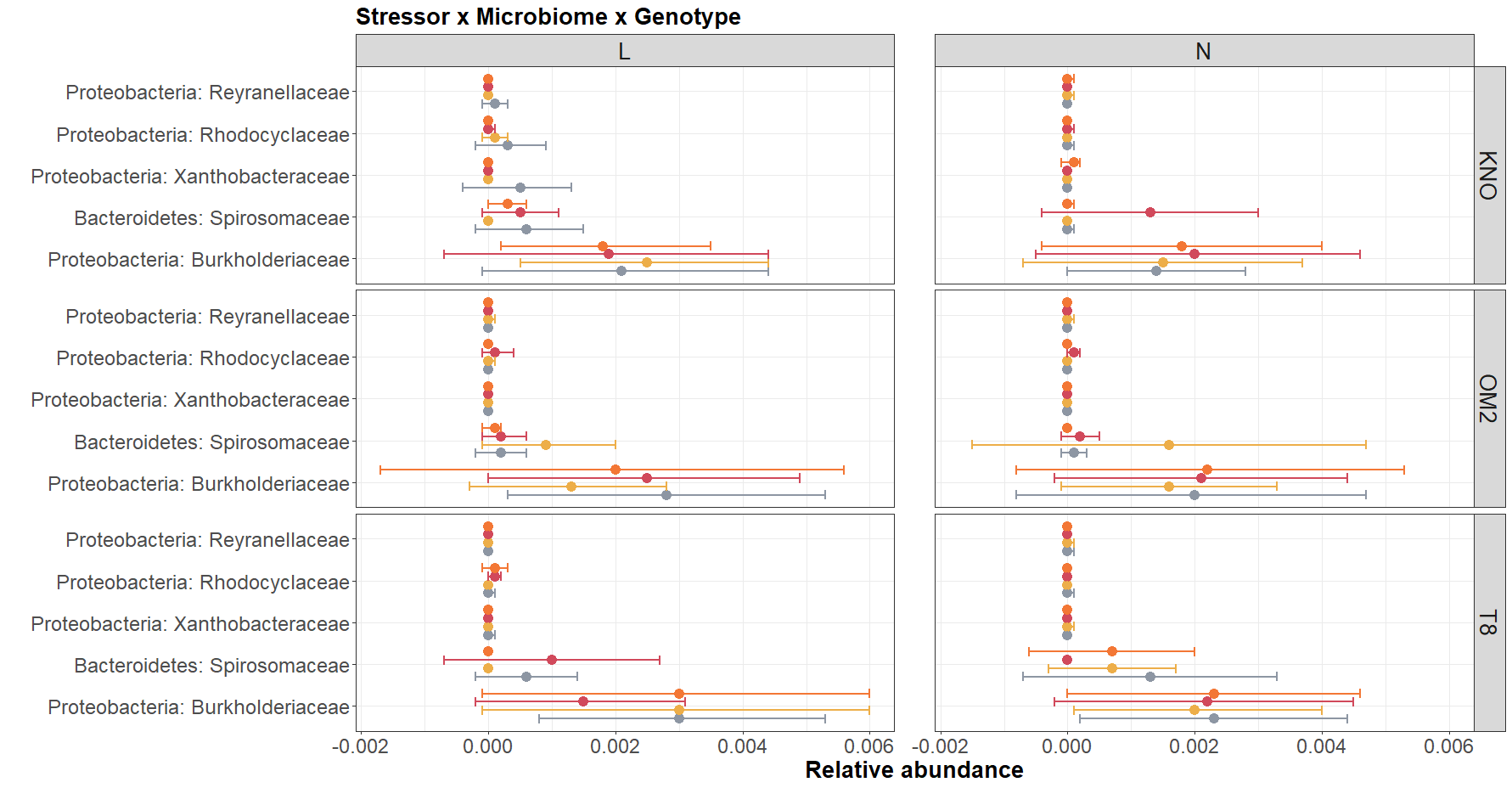 |
| --- |
| 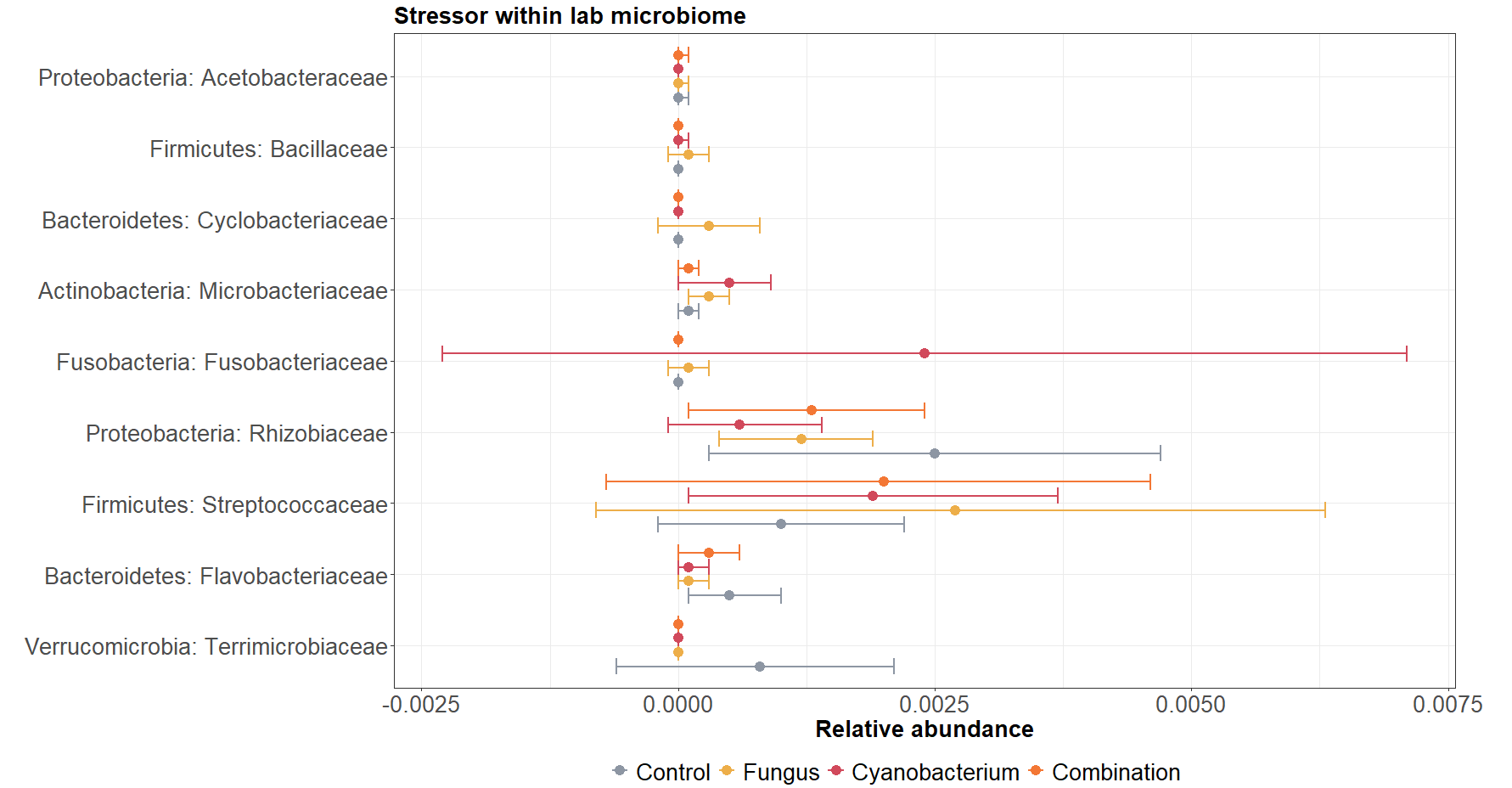 |
| **Figure SI6**: ggplot representing the ASVs at family level that were significantly different between the stressor x microbiome x genotype interaction. Colors indicate the stressor treatments: Grey= control treatment, Yellow= *Aspergillus* infection, Red= *Microcystis* infection, Orange= Combination treatment with both *Aspergillus* and *Microcystis* infection. |

| 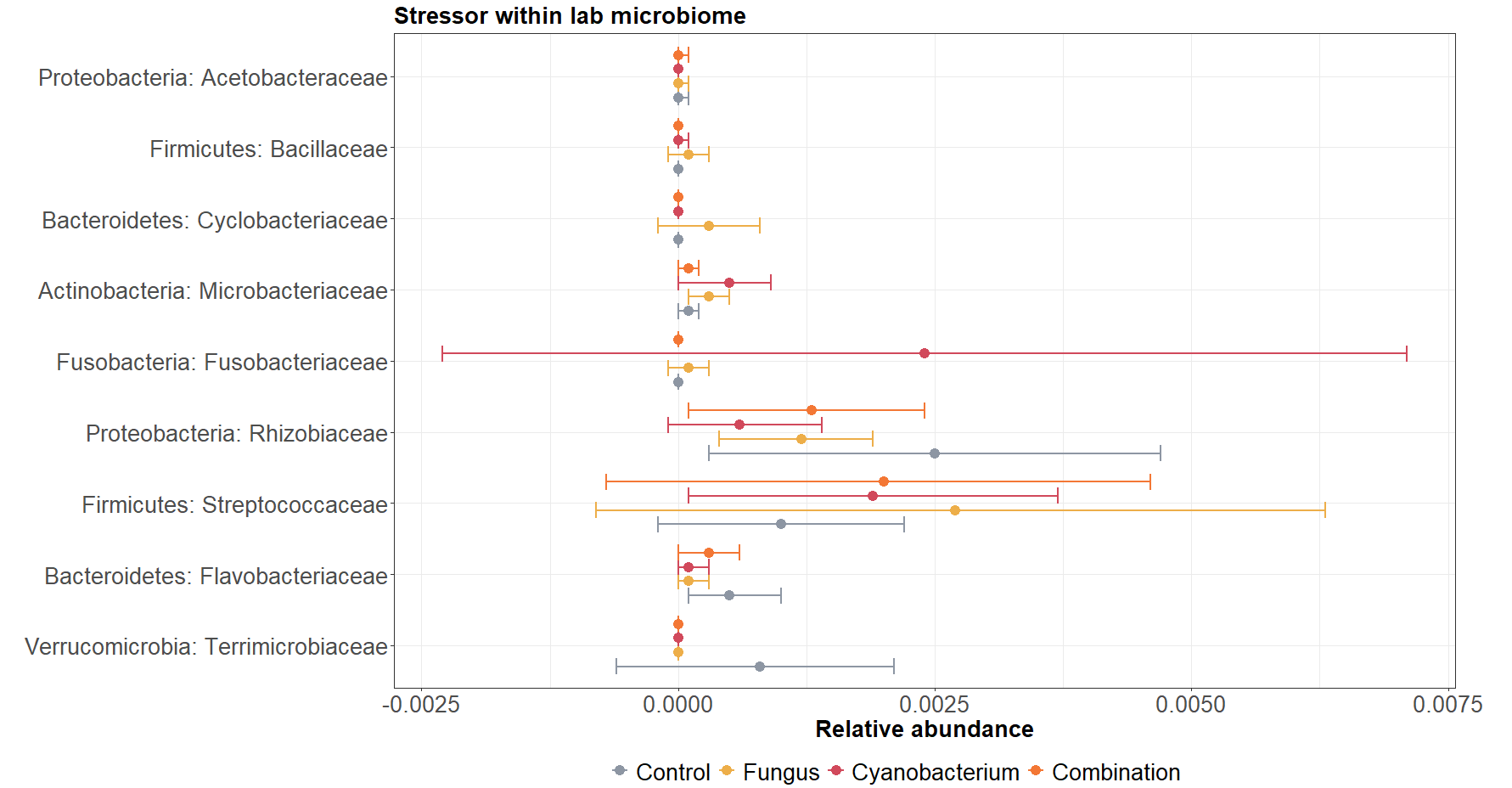 |
| --- |
| A |
| 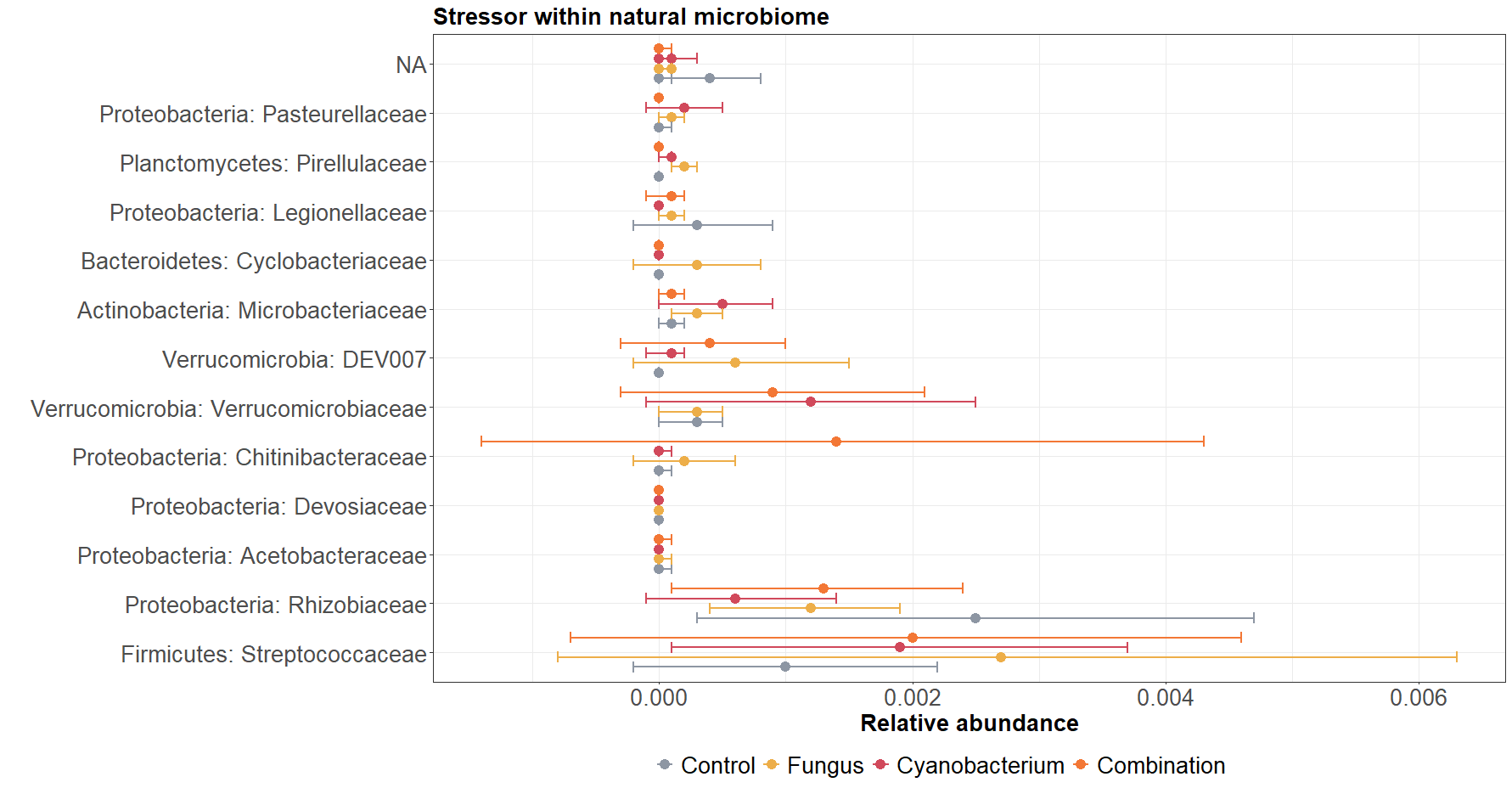 |
| 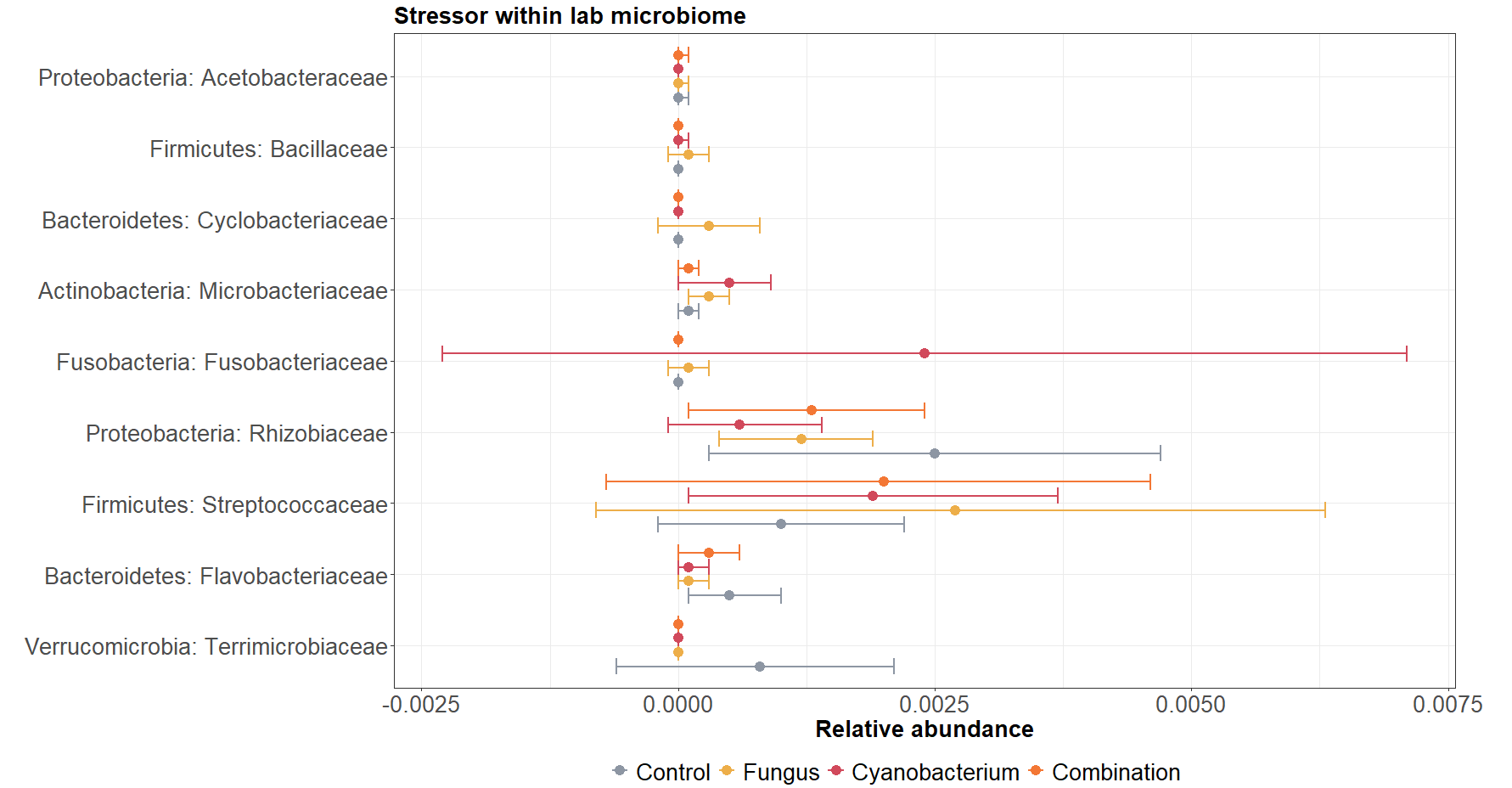 |
|  |
| B |

**Figure SI7:** ggplot representing the ASVs at family level that were significantly different between the different stressor treatments within the (A) lab and (B) natural microbial water treatment. Colors indicate the stressor treatments: Grey= control treatment, Yellow= *Aspergillus* infection, Red= *Microcystis* infection, Orange= Combination treatment with both *Aspergillus* and *Microcystis* infection

**Supplementary tables**

**Table SI3**: Overview of the number of pooled recipient guts per microbial sample. Green: samples in the dataset. Orange: samples lost due to rarefaction. Red: samples lost during molecular work.

**Table SI4**: Overview results for Pearson correlation between the number of guts per sample and ASV richness. Raw and adjusted (adjusted for multiple comparisons through the control of the false discovery rate (FDR)) p-values are given. Significant results (p<0.05) are indicated with *. Highly significant results (p<0.001) are indicated with ***.

|  | **df** | **ASV richness** | | | |
| --- | --- | --- | --- | --- | --- |
|  |  | **cor** | **t** | ***P* value** | **Adjusted *P* value** |
| **Stressor** | 2 | -0.9305785 | -3.5948 | 0.06942 | 0.38185 |
| **Microbiome** | - | - | - | - | - |
| **Genotype** | 1 | -0.560576 | -0.67694 | 0.6212 | 0.62120 |
| **Stressor x Microbiome** | 6 | -0.6089163 | -1.8803 | 0.1091 | 0.38185 |
| **Stressor x Genotype** | 10 | -0.3261138 | -1.0909 | 0.3009 | 0.46095 |
| **Microbiome x Genotype** | 4 | -0.4297461 | -0.95187 | 0.3951 | 0.46095 |
| **Stressor x Microbiome x Genotype** | 21 | -0.2100054 | -0.98432 | 0.3362 | 0.46095 |
| **Stressor x Microbiome x Genotype x Replicate** | 40 | -0.1449094 | -0.92626 | 0.3599 | 0.46095 |

**Table SI5**: Survival analysis, significant four-way interaction (infection x cyanobacterium x microbiome x genotype) split up per fixed factor. Per fixed factor all interactions were further investigated. Significant results (p<0.05) are indicated with *. Highly significant results (p<0.001) are indicated with ***.

**Table SI6**:Fecundity analysis, significant four-way interaction (infection x cyanobacterium x microbiome x genotype) split up per fixed factor. Per fixed factor all interactions were further investigated. Significant results (p<0.05) are indicated with *. Highly significant results (p<0.001) are indicated with ***.

**Table SI7**:Post-hoc body size. Difference between different stressor treatments. Significant results (p<0.05) are indicated with *. Highly significant results (p<0.001) are indicated with ***.

| **Contrast** | **Estimate** | **SE** | **Df** | **t-ratio** | ***P* value** |
| --- | --- | --- | --- | --- | --- |
| Control – Infection | 0.10 | 0.02 | 204 | 4.21 | 0.0002*** |
| Control – Cyano | 0.22 | 0.02 | 204 | 8.94 | <0.0001*** |
| Control – Combi | 0.27 | 0.02 | 204 | 10.91 | <0.0001*** |
| Infection – Cyano | 0.11 | 0.02 | 204 | 4.64 | <0.0001*** |
| Infection – Combi | 0.16 | 0.02 | 204 | 6.62 | <0.0001*** |
| Cyano – Combi | 0.05 | 0.02 | 204 | 2.03 | 0.18 |

**Table SI8**: Overview orders in the gut microbial samples of the recipients and donors. Mean and standard deviation (STDV) are given for the relative abundance per order.

**Table SI9**: Results of post-hoc analyses on donor and donor + recipient ASV richness and beta diversity for the significant results of the statistical analysis. Raw and adjusted (adjusted for multiple comparisons through the control of the false discovery rate (FDR)) p-values are given for the post hoc analyses on beta diversity. Significant results (p<0.05) are indicated with *. Highly significant results (p<0.001) are indicated with ***.

|  |  |  | **df** | **ASV richness** | | **Beta diversity** | | |
| --- | --- | --- | --- | --- | --- | --- | --- | --- |
|  |  |  |  | ***P* value** | **z-value** | ***P* value** | **Adjusted *P* value** | ***R*^2^** |
| **Donor + Recipient** | | | | | | | | |
|  | Sample Type | Donor vs Recipient | 1 | <0.001*** | 9.788 | 0.001*** | 0.0040* | 0.08099 |
|  | Microbial inoculum | Lab vs Natural | 1 | <0.001*** | -7.849 | 0.002* | 0.0040* | 0.06912 |
| Sample Type x Microbial inoculum | Within Donor | Lab vs Natural | 1 | <0.001*** | -6.395 | 0.1 | 0.1000 | 0.34698 |
|  | Within Recipient | Lab vs Natural | 1 | <0.001*** | -4.701 | 0.003* | 0.0045* | 0.08844 |
|  | Within Lab | Donor vs Recipient | 1 | 0.0009* | 3.778 | 0.022* | 0.0264* | 0.10831 |
|  | Within Natural | Donor vs Recipient | 1 | <0.001*** | -8.686 | 0.002* | 0.0040* | 0.1609 |
|  |  | Recipient Lab vs Donor Natural | 1 | <0.001*** | -15.640 |  |  |  |
|  |  | Recipient Natural vs Donor Lab | 1 | 0.6432 | 1.174 |  |  |  |
| **Donor** | | | | | | | | |
|  | Microbial inoculum | Lab vs Natural | 1 | <0.001*** | -6.395 |  |  |  |

**Table SI10**: Results of the Pearson Correlation tests between and within life history traits and ASV richness of the gut microbial community. Significant results (p<0.05) are indicated with *. Highly significant results (p<0.001) are indicated with ***.

|  | **DF** | **cor** | **t** | ***P* valus** | **Adjusted *P* value** |
| --- | --- | --- | --- | --- | --- |
| **Survival & ASV richness** | 40 | -0.01893206 | -0.11976 | 0.9053 | 0.905300 |
| **Fecundity & ASV richness** | 40 | -0.02058545 | -0.13022 | 0.897 | 0.905300 |
| **Body Size & ASV richness** | 40 | 0.05825961 | 0.36909 | 0.714 | 0.905300 |
| **Survival & Fecundity** | 70 | 0.3216004 | 2.8417 | 0.005875* | 0.017625* |
| **Survival & Body Size** | 70 | 0.1668733 | 1.416 | 0.1612 | 0.332400 |
| **Fecundity & Body Size** | 70 | 0.3343081 | 2.9678 | 0.004103* | 0.017625* |

**Table SI11**: Overview significantly different OTUs after edgeR analysis (p<0.001 for all variables except for the three-way interaction in the recipients (p<0.05)).
